# A Randomised, Controlled Trial: Effect of a Multi-Strain Fermented Milk on the Gut Microbiota Recovery after *Helicobacter pylori* Therapy

**DOI:** 10.3390/nu13093171

**Published:** 2021-09-11

**Authors:** Eric Guillemard, Marion Poirel, Florent Schäfer, Laurent Quinquis, Caroline Rossoni, Christian Keicher, Frank Wagner, Hania Szajewska, Frédéric Barbut, Muriel Derrien, Peter Malfertheiner

**Affiliations:** 1 Danone Nutricia Research, Department of Innovation Science and Nutrition, 91767 Palaiseau, France; poirel.marion@gmail.com (M.P.); florent.schafer@danone.com (F.S.); laurent.quinquis@danone.com (L.Q.); caroline.rossoni@outlook.fr (C.R.); muriel.derrien@danone.com (M.D.); 2 Charité Research Organisation GmbH, 10117 Berlin, Germany; christian.keicher@charite-research.org (C.K.); frank.wagner@charite-research.org (F.W.); 3 Department of Paediatrics, Medical University of Warsaw, 02-091 Warszawa, Poland; hania@ipgate.pl; 4 INSERM S-1139, Université de Paris, 75006 Paris, France; frederic.barbut@aphp.fr; 5 Department of Gastroenterology, Hepatology and Infectious Diseases, Magdeburg Clinic, OVGU University, 39120 Magdeburg, Germany; peter.malfertheiner@med.ovgu.de; 6 Department of Internal Medicine II, LMU University Clinic, 81377 München, Germany

**Keywords:** antibiotics, *Helicobacter pylori*, antibiotic associated diarrhea, gut microbiota, short chain fatty acids, recovery

## Abstract

*Helicobacter pylori* (*Hp*) eradication therapy alters gut microbiota, provoking gastrointestinal (GI) symptoms that could be improved by probiotics. The study aim was to assess the effect in *Hp* patients of a Test fermented milk containing yogurt and *Lacticaseibacillus* (*L. paracasei* CNCM I-1518 and I-3689, *L. rhamnosus* CNCM I-3690) strains on antibiotic associated diarrhea (AAD) (primary aim), GI-symptoms, gut microbiota, and metabolites. A randomised, double-blind, controlled trial was performed on 136 adults under 14-day *Hp* treatment, receiving the Test or Control product for 28 days. AAD and GI-symptoms were reported and feces analysed for relative and quantitative gut microbiome composition, short chain fatty acids (SCFA), and calprotectin concentrations, and viability of ingested strains. No effect of Test product was observed on AAD or GI-symptoms. *Hp* treatment induced a significant alteration in bacterial and fungal composition, a decrease of bacterial count and alpha-diversity, an increase of *Candida* and calprotectin, and a decrease of SCFA concentrations. Following *Hp* treatment, in the Test as compared to Control group, intra-subject beta-diversity distance from baseline was lower (*p*_adj_ = 0.02), some *Enterobacteriaceae*, including *Escherichia-Shigella* (*p*_adj_ = 0.0082) and *Klebsiella* (*p*_adj_ = 0.013), were less abundant, and concentrations of major SCFA (*p* = 0.035) and valerate (*p* = 0.045) were higher. Viable *Lacticaseibacillus* strains were detected during product consumption in feces. Results suggest that, in patients under *Hp* treatment, the consumption of a multi-strain fermented milk can induce a modest but significant faster recovery of the microbiota composition (beta-diversity) and of SCFA production and limit the increase of potentially pathogenic bacteria.

## 1. Introduction

Antibiotics have been reported to alter gut microbiota to variable extents, which may lead to an increased prevalence of opportunistic pathogens, such as *Clostridioides difficile (Cd)*, a decrease of the metabolism of primary bile acids and of non-digested carbohydrate, and a reduction of short-chain fatty acids (SCFA) production [1]. Also non-antibiotics drugs, like proton pump inhibitor (PPI), can alter the gut microbiome [2]. As a consequence, a series of gastrointestinal (GI) symptoms, including antibiotic associated diarrhea (AAD), may result [1,3], and be responsible for treatment discontinuation and induction of antibiotic resistance, as reported in the case of *Helicobacter pylori* (*Hp*) eradication treatment [3].

The recent definition of *Hp* gastritis as an infectious disease has extended the indication for therapy independent of the presence of symptoms or clinical complications [4,5]. In regions with low clarithromycin resistance (<15%), PPI-based standard triple treatment for 14 days with clarithromycin and amoxicillin (or metronidazole) is a recommended first-line therapy for *Hp* eradication [5,6]. Several studies reported that *Hp* treatments, including standard triple therapy, can induce alteration of gut microbiota that might persist for several months [7], with associated GI-symptoms including diarrhea in some studies [8,9]. However, no study took the loss of microbial load into account, allowing a quantitative microbiome profiling [10]. The individual effects of amoxicillin or metronidazole in reducing the production of SCFA were reported [11,12] but the effect of complete *Hp* treatment has never been assessed. Also, the effects of *Hp* treatment on gut microbiota (bacteria and fungi), SCFA, and associated side effects have never been investigated within the same trial.

Probiotics are defined as live microorganisms that, when administered in adequate amounts, confer a health benefit on the host. Health benefits provided by probiotics were shown on diverse clinical endpoints, including gastrointestinal ones such as infectious diarrhea, AAD, gut transit, IBS, abdominal pain and bloating, ulcerative colitis, and necrotizing enterocolitis [13]. The benefit of probiotics in combination with *Hp* treatment regimen is, however, uncertain [3]. Meta-analyses reported that probiotics [14], either a specific strain [15] or multi-strain combinations [16], can improve *Hp* eradication rate and reduce *Hp* treatment side effects, including diarrhea. Additionally, intake of probiotics helped to reduce gut microbiota disruption induced by *H. pylori* eradication therapy [1].

A dairy product containing the strain *Lacticaseibacillus paracasei* (*Lp*) CNCM I-1518 was previously shown to reduce both antibiotic associated diarrhea (AAD) and *Cd*-associated diarrhea (CDAD) occurrence in hospitalized elderly [17,18] and to increase the *Hp* eradication rate in children [19]. Recently, a seven-strain fermented milk product containing *Lp* CNCM I-1518, *Lp* CNCM I-3689, *Lacticaseibacillus rhamnosus* (*Lr*) CNCM I-3690, and yogurt strains, was shown to be safe in healthy subjects [20].

The objective of the present trial was to assess the effect of four-week consumption of this multi-strain product, on AAD and GI-symptoms, on the gut microbiota composition, and on the SCFA and calprotectin production, in a population of adult dyspeptic patients treated for *Hp* eradication.

## 2. Material and Methods

### 2.1. Study Design

**Figure 1:**
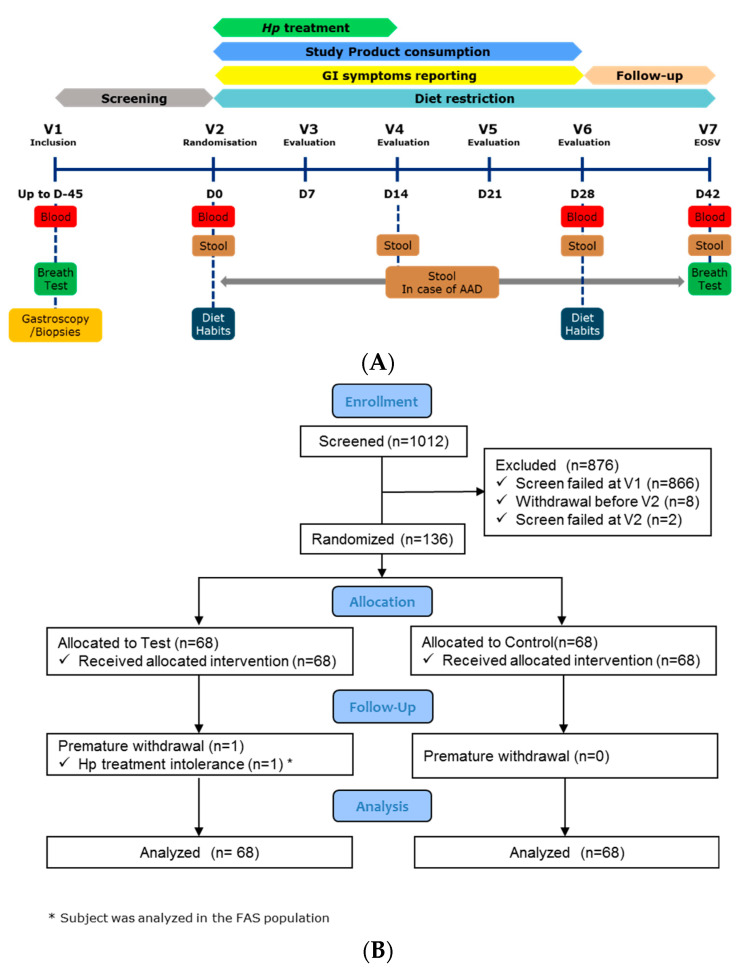
(**A**) Study Design Overview. (**B**) Subject flowchart.

The study was monocentric, randomised, double blind, controlled, with two parallel arms (Test/Control, allocation ratio 1:1) and an adaptive design with interim analysis. As described in Figure 1A, the study included a screening phase, 14-days of *Hp* eradication treatment (D0-D14), 28-days of product consumption (D0-D28) and 14-days of follow-up (D28-D42), with dietary restriction (D0-D42) (no yogurts, probiotics in fermented dairy products, or supplements). Seven visits were planned, in a clinical unit (Charité Research Organisation GmbH, Berlin, Germany): for inclusion (V1), randomisation (V2-D0), and evaluation (V3-D7 to V7-D42), with blood (fasted) and stool sampling as described in Figure 1A and Supplementary Materials. Additional stools were collected at the first day and after the end of each AAD episode. The study was performed in accordance with the Declaration of Helsinki and Good Clinical Practice (International Conference on Harmonisation E6) and approved by the Ethics Committee of Charité–Campus Mitte (Application Number EA1/297/15) of the Charité–Universitätsmedizin Berlin, Germany. All volunteers provided a signed informed consent form (ICF). The trial was registered on clinicaltrials.gov (registration number: NCT02900196).

### 2.2. Subject Selection

Screening lasted from 16 September 2016 (first inclusion at ICF signature) to 31 May 2017 (last inclusion), and the study experimental phase from 7 October 2016 (first randomisation) to 10 August 2017 (last visit).

Main inclusion criteria were: *Hp* infection, based on positive ^13^C-Urea Breath test and at least one positive Urease or *Hp* gastritis histological test; dyspepsia with medical prescription for a *Hp* eradication triple therapy (pantoprazole, clarithromycin, and amoxicillin for 14 days); age 18 to 65 years and a body mass index (BMI) of 19 to 30 kg/m².

Main exclusion criteria were: past *Hp* eradication treatment; alarm feature (bleeding, anemia, unexplained weight loss, dysphagia, odynophagia, recurrent vomiting, or previous gastrointestinal malignancies); benign peptic ulcer, pre-malignant or malignant lesion; diarrhea within the preceding four weeks; severe evolutive, chronic, or past pathology or infection of the GI-tract or surgery in the last three months; liver or renal diseases (based on blood transaminase and creatinine); antibiotics, intestinal antiseptic treatment during the previous two months or chronic use of laxatives or anti-diarrheal; H2-receptor antagonists or PPI treatment in the last two weeks or food allergy. Decision tree applied for screening to select *Hp* infected dyspeptic subjects without alarm symptoms or specific gastric pathologies is presented in Supplementary Figure S1. All criteria, including laboratory results from the screening period, were checked again at randomisation. Eligibility criteria are detailed in the Supplementary Materials.

### 2.3. Product Intervention and Hp Eradication Treatment

Test product was a fermented milk containing *Lp* CNCM I-1518, *Lp* CNCM I-3689, and *Lr* CNCM I-3690 strains and four yogurt strains. Strain counts are provided in Supplementary Table S1. Control product was an acidified milk, depleted in lactose, containing phosphoric acid, and Carboxy Methyl Cellulose. Both products were manufactured by Danone Research, France, and were similar in sweetness, flavour (multi-fruit), texture, colour, packaging, and nutritional content (isocaloric) to ensure the double-blinding for participants and study personnel including the outcome assessors. Subjects ingested two bottles (100 g/bottle) of Test or Control product per day (one at breakfast, one at dinner), for 28 days.

For *Hp* eradication, subjects were treated by a triple therapy (ZacPac^®^, Takeda, Singen, Germany) including a PPI (pantoprazole 40 mg), and two antibiotics (clarithromycin 500 mg and amoxicillin 1000 mg), twice daily, for 14 days.

### 2.4. Clinical Outcomes

The primary outcome was the occurrence of AAD (number of subjects presenting at least one AAD episode) during the 28-day product consumption period. AAD was defined as three or more loose or liquid stools at 5 to 7 in Bristol Stool Scale (BSS) per day for at least three consecutive days (definition 1 for main analysis) or for at least one day (definition 2 as secondary analysis of the primary outcome), in line with diarrhea definition provided by WHO [21] and previous studies [17,18]. Secondary outcomes included: AAD occurrence with alternative definitions (two or more BSS 5-7 stools per day for at least one or three days; Three or more BSS 5-7 stools per day for at least two days or more than 6 BSS 5-7 stools for at least one day); AAD duration; time to event of AAD; occurrence, duration, or time to event of CDAD (AAD with a positive test for *Cd,* method is provided in Supplementary Materials); cumulative number of days with GI-symptoms (diarrhea, abdominal pain, bloating, nausea, or vomiting); score of Gastrointestinal Symptom Rating Scale (GSRS) questionnaire (based on a 7-graded Likert scale). Monitored safety parameters were vital signs, anthropometry, blood analyses, and adverse events (AE). Details for safety parameter analyses are provided in Supplementary Materials.

### 2.5. Biological Outcomes

For microbiota composition and quantification measures, a total of 558 fecal samples was collected in the study from 135 (67 in Test and 68 in Control group) subjects at four time points (Figure 1A). All samples were then processed for total bacterial count (flow cytometry) and for DNA extraction in view of gut microbiota profiling (bacterial and fungal communities). 16S rRNA gene was sequenced using V3-V4 primers for 16S rRNA gene and ITS2. Short and branched chain fatty acids (SCFA), further categorized as major (acetate, propionate, and butyrate) and minor (valerate, caproate, isobutyrate, and isovalerate), calprotectin, and total and viable *Lp* and *Lr* strains from Test product were analyzed in fecal samples from a subgroup of subjects, SCFA were also quantified in blood. Full methods are described in Supplementary Materials.

### 2.6. Procedure

Subjects reported their physical activity and smoking habits (V2), dietary habits (V2 and V6), and alcohol consumption (all visits). In an e-diary, they reported daily their compliance (to *Hp* treatment, product consumption, and dietary restrictions), AE, medication or supplement intake, all passages of stool and consistency (BSS), GI-symptoms, and weekly reported their GSRS scores. ^13^C-Urea Breath test was done at V1 for *Hp* infection diagnosis and V7 for eradication measure. The study was performed in accordance with the protocol with no change during the study. Subject follow-up and detailed procedures are provided in Supplementary Materials.

### 2.7. Sample Size

Sample size was computed based on an expected reduction of 50% (RR = 0.5) of the number of subjects experiencing AAD, a 15% AAD occurrence in the Control group and a 10% drop-out rate [22,23]. A total of 295 randomised subjects was initially planned. After an interim analysis, the study was stopped due to futility of clinical outcomes and the final sample size was 136 randomised subjects. Interim analysis process is described in Supplementary Materials.

### 2.8. Randomisation

A permuted block randomisation (1:1 ratio) was made using an Interactive Web Response System. The randomisation list was generated by an external statistician and kept confidential at the sponsor’s premise to ensure allocation concealment. Subjects were automatically assigned to the Test or Control group. The allocation was blinded to investigator and sponsor. An independent access was provided to the technician responsible for product preparation.

### 2.9. Statistical Analysis

#### 2.9.1. Clinical Outcomes

Analyses were performed using SAS 9.4 software on the Full Analysis Set (FAS) population. The primary and secondary outcomes were described by product group and by visit. Due to the low number of AAD observed, no statistical tests were performed on related outcomes. A descriptive analysis of the number of days with GI-symptoms and of GSRS scores (total, individual, and by symptom dimension scores and their change from V2) was performed.

#### 2.9.2. SCFA, Calprotectin, and Quantification of Test Product Strains in Feces

The effect of product (Test, Control) was assessed on SCFA, calprotectin and their changes from V2, V4, and V6, using a repeated linear mixed model including the values at baseline, the visit, and a Group-Visit interaction as covariates. For Test product strains analysis, the effect of different time points was assessed using a Friedman (for total and viable cell count) and a Wilcoxon test (for viability loss and rate). More details are provided in Supplementary Materials.

#### 2.9.3. Gut Microbiota

Statistical analyses were performed, and graphs were plotted with R software (version 3.6.0). Total bacterial count (as log_10_-transformed), alpha diversity, Shannon index ITS/16S ratio were analyzed as change from baseline using linear mixed model (nlme 3.1–140) as described above or using Mann–Whitney or Wilcoxon signed rank test when normality was rejected. Beta-diversity was analyzed using PERMANOVA (vegan 2.5–6) and pairwise group mean dispersions using Tukey HSD. Quantitative Microbiome Profiling (QMP) was done as described by Vandeputte et al. [10]. Differential analyses were performed with DESeq2 (version 1.24.0) on 16S based QMP and ITS datasets. Fold changes were evaluated with the Wald test (FDR adj. *p* < 0.1). *p*-values were adjusted for multiple testing with the Benjamini–Hochberg procedure (*p*_adj_). Methods are detailed in the Supplementary Materials.

## 3. Results

### 3.1. Subject Enrollment, Population at Baseline, and Compliance

Of the 1012 subjects included and screened, 136 subjects (FAS population) were randomised (68 in each group) (Figure 1B). One subject withdrew from the Test group before V3 due to *Hp* treatment intolerance but was included in the FAS.

Subject characteristics at baseline (Table 1) were well-balanced between groups for age, sex, BMI, physical activity (IPAQ), and medical or surgical history. Proportions of alcohol consumers and smokers were slightly higher in the Test group whereas smokers in the Control group consumed a higher number of cigarettes per week. A higher proportion of subjects with concomitant medication, mainly oestrogens/progestogens and anti-inflammatory medication, was reported in the Test group. Reporting of dietary habits (FFQ) showed no difference between groups at V2 and V6 in terms of diet total energy and water content and intake of nutrients, including protein, fat, carbohydrates, short chain fatty acids, minerals, vitamins, and fibers. The observed differences in baseline characteristics were considered to have no expected impact on study product effect evaluation.

Subject compliance (mean (SD)) was high in both Test and Control groups for study product consumption (99.2 (3.3) % and 99.7 (1.0) %) and for *Hp* treatment (97.2 (12.2) % and 98.4 (7.7) %). Major deviations to the protocol were reported in only 14 (10.3%) subjects (seven in each group), mainly due to low compliance to *Hp* treatment or study products consumption. A small amount of missing data was reported for clinical parameters (<10%).

### 3.2. Clinical Outcomes

At V7, *Hp* eradication was successful in 83.8% and 88.2% of subjects in Test and Control groups, respectively. As primary outcome, the occurrence of AAD was much lower than expected since only three and eight subjects reported an episode as per definition 1 and 2, respectively, in the FAS population (Table 2). All AAD episodes were reported during the *Hp* treatment period. Due to the limited number of episodes, no statistical test could be applied for the main analysis of the primary outcome or for any other clinical outcomes consistent with the planned strategy to handle multiple testing. All clinical criteria were analyzed based on descriptive statistics. The limited number of events did not allow any conclusion on the product efficacy on AAD occurrence (Table 2). No CDAD was reported and only two *Cd* positive subjects were found in the Test group, likely as a consequence of *Hp* treatment (Table 2). No relevant difference between groups was observed for other clinical outcomes, as described in Supplementary Files (Supplementary Materials, Table S2, Figure S2), including occurrence of AAD with alternative definition, time to first event, AAD duration, number of days with GI-symptoms, or GSRS scores.

### 3.3. Test Product Strains Viability in Feces

The viability of the three strains *Lp* CNCM I-3689, *Lr* CNCM I-3690, and *Lp* CNCM I-1518 was assessed in the feces of 48 subjects (25 in Test, 23 in Control groups) (Supplementary Figure S3). At V4, total cell (live and dead) counts reached 8.1 to 8.3 log_10_ cells/g feces depending on the strain with a viability rate (i.e., % of total cell counts) of 2.2% (*Lr* CNCM I-3690), 2.3% (*Lp* CNCM I-3689), and 9.4% (*Lp* CNCM I-1518). At V6, no significant change was observed for either the total or viable cell counts. At V7, two weeks after the end of the last Test product intake, both total and viable bacteria count significantly decreased for all three strains as compared to V6 (Wilcoxon signed-rank, *p* < 0.001) to an undetectable level (LOQ), except for *Lr* I-3690 in five and two subjects (20% and 8% of subjects) and for *Lp* I-3689 strain in one and one subject (4% and 4% of subjects), respectively. Additional data are provided in Supplementary Materials.

### 3.4. Global Gut Microbiota Response to Hp Treatment and Product Intervention

**Figure 2:**
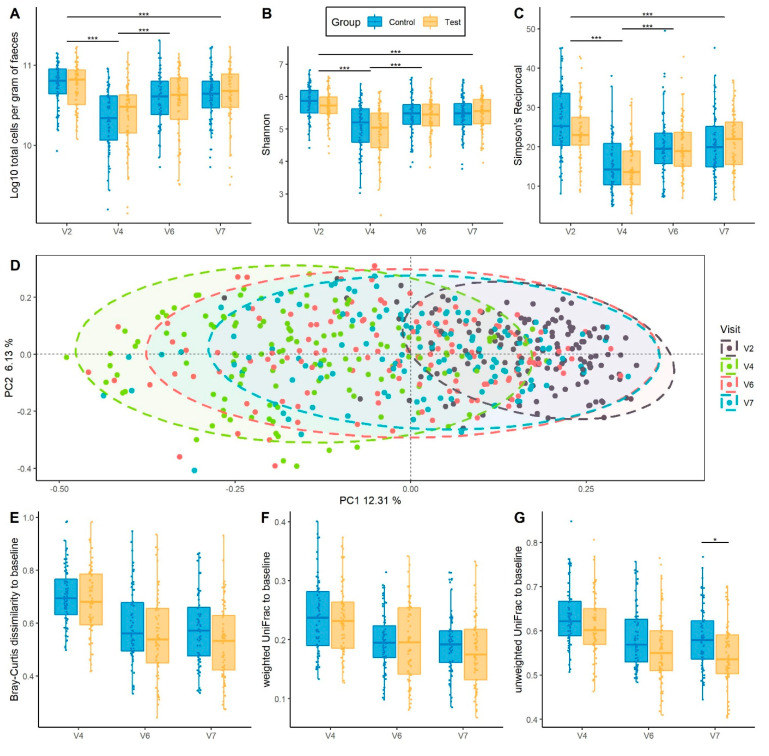
Global gut microbiota response to *Hp* treatment and product intervention. (**A**) Total bacterial count/g fecal samples assessed by flow cytometry, with a log_10_ transformation. (**B**) Alpha-diversity assessed by Shannon index (**C**) Alpha-diversity assessed by Simpson’s Reciprocal. (**D**) Principal Coordinate Analysis (PCoA) based on Bray–Curtis dissimilarity. Samples were collected before (V2) and after *Hp* treatment (V4), 14 days (V6) and 28 days (V7) following cessation of *Hp* treatment. * *p* < 0.05 and *** *p* < 0.001 according to linear mixed model. (**E**–**G**) Intra-subject distance to baseline of each subject in Test and Control groups across the study (**E**) Bray–Curtis dissimilarity (**F**) Weighted UniFrac distance (**G**) Unweighted UniFrac distance. * *p* < 0.05 and *** *p* < 0.001 according to Mann–Whitney test.

Gut microbiota was analyzed in 135 subjects (67 in Test and 68 in Control group) using flow cytometry and 16S rRNA gene sequencing. In both groups, there was a significant loss of total bacteria at V4, after two weeks of products intake and concomitant *Hp* treatment (−0.4 log_10_, *p*_adj_ = 8.66^−24^) (Figure 2A). Bacteria count then gradually increased until V7, 28 days following cessation of *Hp* treatment, to a level lower than that of baseline (−0.13 log_10_, *p*_adj_ = 5.34^−05^). No global difference between Test and Control groups was observed across time points (*p* = 0.64) (Figure 2A). The same kinetics was observed for alpha-diversity indexes, assessed by Shannon and reciprocal Simpson, which decreased at V4 (*p*_adj_ = 3.19^−28^ and *p*_adj_ = 3.08^−32^, respectively), and gradually increased without reaching baseline level at V7 (*p*_adj_ = 1.25^−07^ and *p*_adj_ = 1.39^−10^, respectively), with no difference observed between groups (*p*_adj_ = 0.74 and *p*_adj_ = 0.49, respectively) (Figure 2B,C). Beta-diversity, assessed by Principal Coordinate Analyses (PCoA) for Bray–Curtis dissimilarity, revealed a shift in the global microbiota composition at V4 as compared to baseline (PERMANOVA, *p*_adj_ = 1.25^−3^ (Figure 2D) and a gradual but incomplete recovery at V7 (PERMANOVA, *p*_adj_ = 1.25^−3^). This was also observed with UniFrac distances (Supplementary Figure S4). We observed higher variability in beta-diversity following *Hp* treatment than at baseline (Tukey HSD, *p*_adj_ = 3.31^−10^).

Intra-subject distance to baseline was computed for the three beta-diversity metrics between baseline and each of the following time points. A gradual decrease of intra-subject distance in both groups following cessation of *Hp* treatment was observed with no difference between groups based on Bray–Curtis dissimilarity following correction for multiple testing (Mann–Whitney test, *p* = 0.04 and *p*_adj_ = 0.12 at V7) and Weighted UniFrac (Figure 2E,F). Unweighted UniFrac intra-subject distance tended to be lower in Test group at V4 and V6 (Mann–Whitney test, *p*_adj_ = 0.07 for both) and was significantly lower at V7 (Mann–Whitney test, *p*_adj_ = 0.02) (Figure 2G). Results from alpha and beta-diversity measured by quantitative microbiome profiling (QMP) were consistent with that of relative microbiome profiling (Supplementary Figure S5). In addition, lower intra-subject distance was observed in the Test group at V7 for Bray–Curtis dissimilarity by QMP (Mann–Whitney test, *p*_adj_ = 0.03) (Supplementary Figure S5).

### 3.5. Taxonomical Analysis by Quantitative Microbiome Profiling

**Figure 3:**
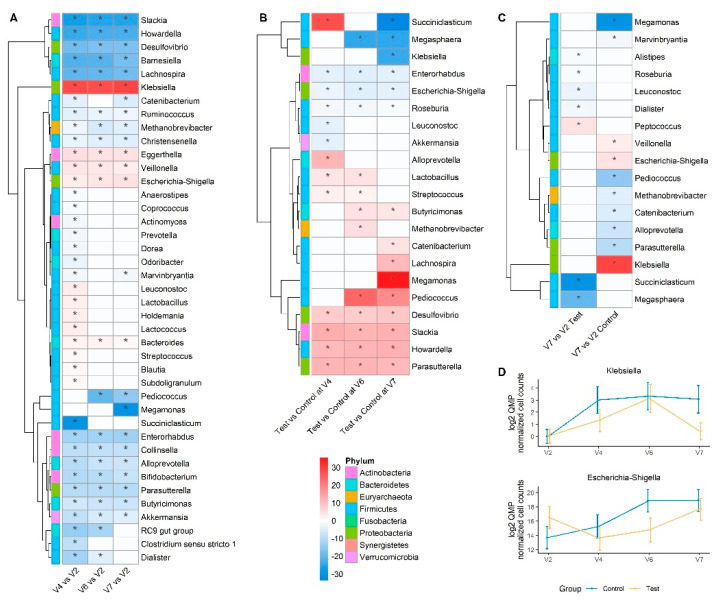
Genus-level differential analysis based on Quantitative Microbiome Profiling (**A**) Heatmap of differentially abundant genera between each visit and baseline (V2) in the Control group. Red indicates higher fold-change and blue lower fold-change with regards to baseline (**B**) Heatmap of differentially abundant genera between Test and Control groups at each visit. Red indicates a higher fold-change and blue a lower fold-change in Test group (**C**) Heatmap of differentially abundant genera between follow-up (V7) and baseline (V2) which behave differently in the two groups. Red indicates higher fold-change and blue lower fold-change with regards to baseline (V2). For all heatmaps, only taxonomically assigned genera were selected based on QMP normalized counts >10.000.000, (FDR adj. * *p* < 0.1, DESeq2-based Wald test). Non-significant fold changes were set to zero for heatmap display and significant fold changes are highlighted by a star. (**D**) Quantitative log_2_ abundance of *Escherichia/Shigella* and *Klebsiella* in response to *Hp* treatment along the study, as mean ±95% CI.

At baseline, the gut microbiota from *Hp* patients was dominated by Gram-positive phyla, Firmicutes (71%), Actinobacteria (18%), followed by Bacteroidetes (7%). DESeq2 analysis based on QMP (205 genera) showed that 14-day *Hp* treatment induced differential quantitative abundance of taxonomically diverse genera in the Control group (see: Supplementary Table S3 for fold change and adjusted *p* values). Genera with depleted abundance following *Hp* treatment included members of Actinobacteria (*Bifidobacterium*, *Collinsella, Slackia*) (Figure 3A), while *Eggerthella, Bacteroides,* and some members from Enterobacteriaceae (*Escherichia-Shigella*, *Klebsiella*) were enriched. Genera whose abundance returned to baseline level included, * Prevotella*, *Coprococcus*, *Dialister*, *Blautia*, *Subdoligranulum* amongst others (Figure 3A and Supplementary Table S3). Bacterial genera whose response differed between Test and Control following *Hp* treatment included *Slackia*, *Desulfovibrio* that were enriched in Test, while *Fusobacterium*, *Coprobacter*, and *Escherichia-Shigella* were enriched in Control (Figure 3B and Supplementary Table S3). Given the difference between groups observed based on beta-diversity, on unweighted UniFrac and Bray–Curtis dissimilarity at V7, we further identified genera whose abundance recovered differently from *Hp* treatment between groups (Figure 3C and Supplementary Table S3). We observed that fewer genera were differently abundant between baseline and V7 in the Test compared to the Control group. Amongst them, *Escherichia-Shigella*, *Klebsiella* (Figure 3C,D), and *Veillonella* were significantly enriched while *Methanobrevibacter* was significantly depleted in the Control but not in the Test group. In contrast, the abundance of some genera, including *Roseburia*, and *Dialister*, was lower at V7 in the Test group but not in the Control.

### 3.6. Gut Mycobiota Response to Hp Treatment and Product Intervention

**Figure 4:**
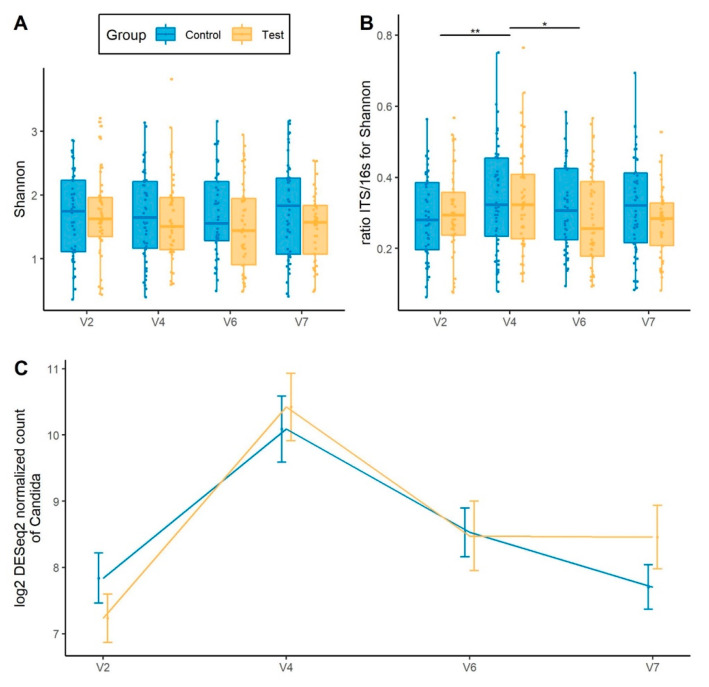
Global gut mycobiota response to *Hp* treatment and product intervention. (**A**) Alpha-diversity assessed by Shannon index (**B**) Shannon index ratio ITS/16S based (**C**) Abundance of *Candida*. * *p* < 0.05 and ** *p* < 0.01 according to Mann–Whitney test.

We monitored fungal dynamics across the study. While there was no change in ITS Shannon index in response to *Hp* treatment (Figure 4A), we observed a transient increase in the fungi to bacteria (ITS/16S) Shannon ratio at V4 (*p*_adj_ = 5.79^−03^) which then reverted to baseline at V7 (*p*_adj_ = 0.864), with a trend for a lower ITS/16S ratio (*p* = 0.08) in the Test group (Figure 4B). The abundance of *Candida* was transiently enriched following 14-day *Hp* treatment (*p*_adj_ = 1.81^−25^), followed by a recovery to baseline level (V2 to V7, *p*_adj_ = 0.467), with no difference between groups (Figure 4C). Additional data are provided in Supplementary Materials and Figure S6).

### 3.7. SCFA and Calprotectin Response to H. pylori Treatment and Product Intervention

**Figure 5:**
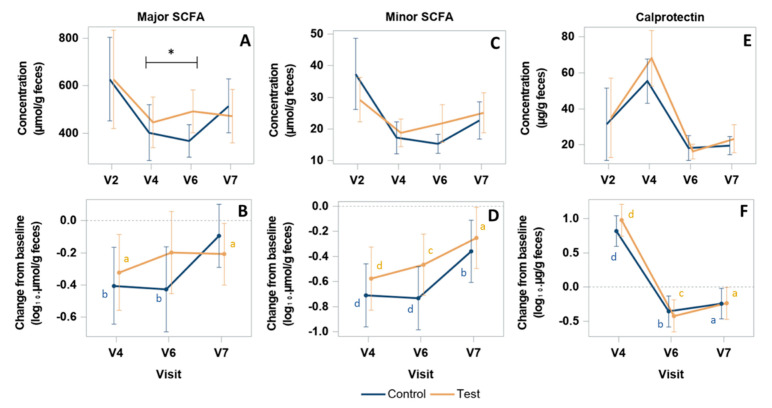
Quantification of fecal major/minor SCFA and Calprotectin. Concentration (**A**,**C**,**E**) and change from baseline (**B**,**D**,**F**) of major SCFA (acetate, propionate, butyrate) (**A**,**B**), minor SCFA (valerate, caproate, isobutyrate, and isovalerate) (**C**,**D**) and Calprotectin (**E**,**F**). *p*-values are provided according to Student test: * *p* < 0.05 for comparison between groups of the change of major SCFA concentration from V4 to V6; ^a^ *p* < 0.05, ^b^ *p* < 0.01; ^c^ *p* < 0.001; and ^d^ *p* < 0.0001, for comparison within group of the change from V2 at each visit. SCFA concentrations are expressed in µmol/g of dry feces.

Major (acetate, propionate, butyrate) and minor (valerate, caproate, isobutyrate, and isovalerate) SCFA were quantified in dry feces from 61 subjects (31 and 30 subjects in Test and Control group, respectively). In both groups, SCFA concentration significantly decreased at the end of *Hp* treatment (V4) as compared to baseline (V2) for major SCFA (*p* = 0.0167 in Test, *p* = 0.0040 in Control) (Figure 5A,B), minor SCFA (*p* < 0.0001 in both groups) (Figure 5C,D), and similarly for each SCFA type (*p* < 0.05) ( Supplementary Figure S7). From V4 to V6, at the end of product consumption, SCFA concentrations globally further decreased in the Control group and systematically increased in the Test group for all SCFA types and categories, albeit with no statistical significance within each group (Figure 5 and Supplementary Figure S7). Evolution in both groups resulted in SCFA concentrations at V6 that were all higher and closer to baseline values in the Test as compared to the Control group. A statistically significant difference between groups was observed in the change from V4 to V6 for major SCFA resulting in concentrations (Mean (SD)) of 492.44 (247.3) and 366.7 (178.6) µmol/g in Test and Control groups, respectively, corresponding to a difference of changes (Mean (95% CI)) of 120.83 (8.21;233.45) µmol/g (Student’s test, *p* = 0.035) (Figure 5A,B). For valerate, a significant difference of change between groups (Mean (95% CI)) of 2.60 (0.06;5.15) µmol/g (Student’s test, *p* = 0.045) was also observed from V2 to V6 (Supplementary Figure S7), corresponding to 32% less decrease of valerate concentration in the Test group. From V6 to V7, two weeks after product consumption ceased, the results showed a trend of stabilization (for major SCFA) or an increase (for minor SCFA) of the concentrations in the Test group and a systematic increase of both in the Control group. This resulted in a similar non statistically different levels of all SCFA concentrations between groups at V7. As compared to baseline, concentrations at V7 were mostly lower, with a significant statistical difference for major SCFA in the Test group and for minor SCFA in both groups (Figure 5 and Supplementary Figure S7). Serum contents of SCFA showed no difference between groups at any timepoint between V2 and V6 (data not shown). Fecal calprotectin was quantified in 73 subjects (35 and 38 subjects in Test and Control groups, respectively). Calprotectin concentration evolved similarly in both groups with a significant increase from baseline (V2) at the end of *Hp* treatment (*p* < 0.0001) (Mean (SD): 68.1 (44.8) and 55.4 (37.3) µg/g at V4, in Test and Control, respectively), followed by a decrease at V6 (*p* < 0.0001), to reach a similar level between groups, below the baseline values (*p* < 0.05) at V7 (Figure 5E,F).

### 3.8. Safety Outcomes

A similar number of AE were reported by 42 (61,8%) subjects in each group (Supplementary Table S4), mainly headaches, nasopharyngitis, vulvovaginal mycotic infection, dysgeusia, and rash. Very few severe AE and one Serious AE (ankle fracture) were reported in the Control group. AE from which the causality was not established were all qualified as unlikely related to the study products. AE due to *Hp* treatment were reported by 41.2% of the subjects in each group. For all blood safety parameters, weight or vital signs, no relevant evolution was observed during product intake and follow-up periods and no relevant differences between groups were found at baseline or at any other timepoint.

## 4. Discussion

The present study investigated the effect of a fermented milk product containing *Lp* CNCM I-1518, *Lp* CNCM I-3689, and *Lr* CNCM I-3690 and yogurt strains (Test), on the prevention of AAD and GI-symptoms, and on the gut microbiota composition and SCFAs when administered in combination with a standard triple *Hp* eradication therapy.

The Test product did not show a significant effect on AAD occurrence or duration. This can be due to the much lower than expected AAD occurrence observed (1.5% in the Control group instead of the 15% as estimated from former trials) which is most probably due to the young–middle age of a study population with no comorbidities. Other considerations refer to diarrhea not recorded as primary criteria in other studies, or to differences in *Hp* eradication regimens, length of symptom reporting or AAD definitions. The resistance of subjects to *Hp* treatment side effects in our study was also reflected by an absence of GI-symptoms according to GSRS score evolution. In older and more sensitive populations, one of the Test product strains showed an effect in reducing both AAD and CDAD occurrence [17,18] with, however, some exceptions [24]. The rate of *Hp* eradication was not different between the Test and Control groups in our study and was consistent with previous reports [25].

We next assessed the response of gut microbiota to *Hp* eradication therapy and to Test product intervention by relative and quantitative approaches. At baseline, the gut microbiota of *Hp* patients was characterized by a low abundance of *Bacteroidetes* compared to that of Actinobacteria, which might be due to differential composition from healthy subjects [26], and/or potentially to the higher efficacy in lysis of Gram-positive including Actinobacteria with the standardized DNA extraction protocol [27]. We showed that the 14-day *Hp* eradication treatment induced gut microbiota alteration, reflected by a decrease in total bacterial count and taxonomically-wide changes that persisted up to 28 days following cessation of the treatment, in line with previous studies showing that recovery of gut microbiota following *Hp* triple therapy, might take more time [28]. Bacteria genera exhibited variable acute and recovery responses, suggesting that a longer follow-up period would have allowed us to study longer-term resilience. The durable loss of *Bifidobacterium* is consistent with a previous study in which its abundance was still depleted three months following *Hp* triple therapy [29]. Distinct responses were observed within the *Coriobacteriia* class, with *Slackia, Collinsella* being depleted while *Egghertella*, previously reported to be depleted in healthy subjects, was more durably enriched following *Hp* eradication [30].

Intra-subject distance to baseline was lower in subjects consuming the Test product, during the post-*Hp* treatment phase suggesting a gradual faster rate of recovery of gut microbiota. This contrasts with the study from Suez et al., who showed that the fecal microbiota recovery was delayed up to five months by the intake of a mixture of 11 lactic acid bacteria and bifidobacteria strains, following broad-spectrum antibiotics intake (ciprofloxacin and metronidazole) in healthy subjects [31]. This highlights the variability of gut microbiota response to distinct strains in the context of antibiotics treatment, which can also depend on intervention design or antibiotics spectrum [32]. In the present trial, we observed an increase of *Escherichia-Shigella* and *Klebsiella*, that persisted in Control but not in Test group up to 28 days after the end of *Hp* treatment, in line with the anti-pathogenic effects of the Test product strains observed in pre-clinical models [33,34]. Overall, the Test product affected gut microbiota mostly during the recovery phase following *Hp* treatment. Also, the Test product effect seemed more pronounced in the present study in case of microbiota alteration than in healthy subjects not exposed to antibiotics as previously reported [20].

The fungal community was previously shown to bloom following antibiotics treatment [35]. Here, a transient increase in the fungi/bacteria ratio (ITS/16S) following the 14-day *Hp* treatment was observed followed by a reversion towards baseline, which tended to be faster in subjects consuming the Test product. This suggests that the balance of fungi to bacteria was altered probably through additional availability in nutrients and/or direct and indirect effects of some bacteria on the fungal population. Notably, the abundance of *Candida* was transiently increased, in line with a report based on culture method [36].

In our study, fecal concentrations of all SCFA decreased in both groups at the end of *Hp* treatment and beyond in the Control group until the end of product consumption and then increased after two weeks of follow-up, but without complete recovery of baseline levels. In contrast, SCFA concentration in the Test group showed a significantly faster recovery following cessation of *Hp* treatment. This evolution profile was systematically observed for each type of SCFA, despite their distinct metabolic pathways, and reached a statistical significance for total major SCFA and valerate. Consistently, *Oscillibacter,* for which some species have been shown to produce valerate [37], and the butyrate producers *Butyrivibrio* and *Butyricimonas*, were more abundant in the Test group as compared to Control. In the last case, butyrate producers could possibly participate to major SCFA increase even though butyrate increase alone was not statistically significant. These results are also in line with the capacity of the three strains *Lr* CNCM I-3690, *Lp* CNCM I-3689, and CNCM I-1518 to increase SCFA production or specific producers abundance in *in vivo* models [33,34,38]. SCFA were shown to create unfavorable conditions for colitogenic *Enterobacteriaceae* [39] which may explain the lower abundance of *Escherichia-Shigella* and *Klebsiella* in Test group. The absence of modification of SCFA in blood in the present study suggests that the observed variations of fecal SCFA might reflect a change of SCFA production in the colon, rather than a modification of absorption in blood from the gut.

The transient detection of viable bacteria strains in the feces throughout the Test product consumption period shows the capacity of the three strains to survive in the GI-tract. Viability rates in the presence (V4) or absence (V6) of *Hp* eradication therapy indicates that strains survival capacity was not hampered by the treatment. In close conditions, similar counts in total [40] or viable strains [41] was recovered in feces of healthy adults or adults under *Hp* treatment supplemented with milk containing other *Lactobacillus casei* or *rhamnosus* strains [40,41]. In our study, two weeks following cessation of product intake, *Lr* CNCM I-3690 was the most detected in the gut among the analysed strains. Whether its longer detection is related to higher microbiota permissivity and/or specific characteristics of the strain, such as the expression of pili [42], warrants further investigation.

A transient increase of calprotectin was also observed in response to *Hp* treatment in association with gut microbiota alteration. Calprotectin is a marker of gut inflammation that correlates with bacterial infectious diarrhea [43,44]. Increase of calprotectin production was also associated with PPI intake [45] possibly due to bacterial overgrowth as a result of inhibition of acid production in the stomach. In the present study, some genera were durably enriched following *Hp* treatment including pathogens potentially inducing AAD such as *Escherichia-Shigella* and *Klebsiella*, consistently to the transient increase of fecal calprotectin. Calprotectin production was also possibly induced by the observed transient increase of *Candida* that has been reported to trigger immune mediated inflammatory pathways associated to gut diseases [46].

This study has some limitations. First, the low occurrence of AAD did not allow us to observe a product effect on clinical outcomes which limits the conclusion on the clinical relevance of the effects on gut microbiota and SCFA related to gastrointestinal symptoms. The trial was also not designed and specifically powered to detect differences in microbiota composition or metabolites between groups. Finally, subjects without *Hp* treatment were not included which may limit the conclusions as to the cause–effect relationship between medication and the observed modification of study outcomes. The strengths of the study rely on its novelty in assessing the effect of a product in subjects under *Hp* eradication treatment on a range of gut microbiota parameters (using quantitative microbiome profiling), on SCFA production and host reactive inflammatory markers, what is more in a same trial and on a higher sample size as compared to previous studies.

## 5. Conclusions

No effect of Test product was observed on AAD or GI-symptoms which can be explained by the unexpected very low occurrence of event in both the Test and Control groups. Our findings suggest that the Test product may confer a better resilience following a *Hp* eradication treatment, therefore protecting the homeostasis of the gut microbiota and SCFA production, and reduce the abundance of potentially pathogenic bacteria. A confirmatory study investigating the microbiota profile as primary criteria is needed for full demonstration of Test product beneficial effects.

## Figures and Tables

**Table 1:** Subject characteristics at baseline.

		Test (*N* = 68)	Control (*N* = 68)
Age (years)	Mean (SD)	42.1 (10.1)	42.6 (11.3)
	Min; Max	26; 65	23; 64
Sex, *n* (%)	Male	34 (50.0)	35 (51.5)
	Female	34 (50.0)	33 (48.5)
BMI (kg/m²)	Mean (SD)	24.8 (2.9)	25.0 (2.6)
	Min; Max	19.6; 30.0	19.7; 29.5
Alcohol Consumer, *n* (%)	No	17 (25.0)	24 (35.3)
	Yes	51 (75.0)	44 (64.7)
Alcohol units/week ^1^	Mean (SD)	2.2 (1.8)	2.3 (1.4)
	Min; Max	1; 8	1; 7
Smoking status, *n* (%)	Current smoker	19 (27.9)	14 (20.6)
	Ex-smoker	20 (29.4)	20 (29.4)
	Never a smoker	29 (42.6)	34 (50.0)
Number of cigarettes/week ^2^	Mean (SD)	41.2 (39.9)	58.1 (40.8)
	Min; Max	1; 140	1; 105
Physical activity IPAQ, n (%)	Low	8 (11.8)	8 (11.8)
	Moderate	24 (35.3)	22 (32.4)
	High	36 (52.9)	38 (55.9)
Medical/Surgical history, *n* (%)		49 (72.1)	47 (69.1)
Concomitant medication or nutritional supplements, *n* (%)		38 (55.9)	27 (39.7)
Product restricted during the study, *n* (%)		0	0

^1^ In alcohol consumer. ^2^ in current smokers. IPAQ = international physical activity questionnaire.

**Table 2:** Occurrence of AAD and CDAD. Occurrence of AAD and CDAD during the 28-day product consumption period. AAD Definitions: Three or more BSS 5–7 stools per day for at least three days (Definition 1) or at least one day (Definition 2).

	Test (*N* = 68)	Control (*N* = 68)
AAD occurrence—definition 1, *n* (%)	2 (2.9)	1 (1.5)
AAD occurrence—definition 2, *n* (%)	5 (7.4)	3 (4.4)
CDAD occurrence—definition 1 or 2, *n* (%)	0	0
*Cd* positive test at baseline (V2), *n* (%)	0	0
*Cd* positive test between V2 and V6, *n* (%)	2 (2.9)	0

## Data Availability

16S sequences associated with this project were deposited in EMBL under BioProject accession PRJEB37553.

## Supplementary Materials

The following are available online at www.mdpi.com/article/10.3390/nu13093171/s1, **Supplementary Methods:** Supplementary methods, **Supplementary Results:** Supplementary results, **Figure S1:** Decision tree for diagnosis of *Hp* infection and subjects screening, **Figure S2:** GSRS, **Figure S3:** Quantification of Test product strains in feces, **Figure S4:** UniFrac beta-diversity, **Figure S5:** Global gut microbiota response to *Hp* treatment by Quantitative Microbiome Profiling, **Figure S6:** Global gut mycobiota response to *Hp* treatment, **Figure S7:** Quantification of fecal individual SCFA, **Table S1:** Bacterial strain counts in Test product during the shelf life, **Table S2:** Number of days of GI symptoms, **Table S3:** Differential abundance of all genera across the study based on QMP, expressed as Fold change and adjusted *p*-values, **Table S4:** Adverse events.
