# Supplementary material for: A Randomised, Controlled Trial: Effect of a Multi-Strain Fermented Milk on the Gut Microbiota Recovery after Helicobacter pylori Therapy
Source: Nutrients. 2021 Sep 11;13(9):3171. doi: 10.3390/nu13093171 (PMC8466689; doi:10.3390/nu13093171)
Supplement: Supplementary file 1 [file nutrients-13-03171-s001.zip › nutrients-1281154 - Supplementary version 3.pdf]

nutrients

MDPI

Supplementary Materials

A Randomised, Controlled Trial: Effect of a Multi-Strain Fermented Milk on the Gut Microbiota Recovery after *Helicobacter pylori* Therapy

Eric Guillemard [1,*], Marion Poirel [1], Florent Schäfer [1], Laurent Quinquis [1], Caroline Rossoni [1], Christian Keicher [2], Frank Wagner [2], Hania Szajewska [3], Frédéric Barbut [4], Muriel Derrien [1,†] and Peter Malfertheiner [5,6,†]

Supplementary materials:

Supplementary Methods: Supplementary methods
Supplementary Results: Supplementary results
Figure S1: Decision tree for diagnosis of *Hp* infection and subjects screening
Figure S2: GSRS
Figure S3: Quantification of Test product strains in feces
Figure S4: UniFrac beta-diversity
Figure S5: Global gut microbiota response to *Hp* treatment by Quantitative Microbiome Profiling
Figure S6: Global gut mycobiota response to *Hp* treatment.
Figure S7: Quantification of fecal individual SCFA
Table S1: Bacterial strain counts in Test product during the shelf life
Table S2: Number of days of GI symptoms
Table S3: Differential abundance of all genera across the study based on QMP, expressed as Fold change and adjusted *p*-values
Table S4: Adverse events

Supplementary Methods

Subject Eligibility Criteria

Inclusion criteria

- 1: Subjects who read and signed the Study Informed Consent Form.
- 2: Adult male/female [18, 65] years old.
- 3: Subjects with a body mass index (BMI) as follows: $19 \leq BMI \leq 30$ kg/m^2
- 4: Subjects positive for *Helicobacter pylori* (*Hp*) infection and symptomatic due to *Hp* infection.

Diagnosis of *Hp* infection based on positive ^{13}C-Urea Breath test and at least one positive test among Urease test and Histological test for *Hp*-gastritis

Subjects symptomatic due to H. pylori infection were intended here as subjects with at least one or more of the following dyspeptic symptoms:

a) *Bothersome postprandial fullness: Uncomfortably full after regular sized meal, more than 1 day/week.*
b) *Early satiation: Unable to finish regular sized meal, more than 1 day/week.*
c) *Epigastric pain: Pain or burning in middle of abdomen, at least 1 day/week.*

Histology of gastric biopsies were performed in positive 13C-Urea Breath test patients with dyspeptic symptoms

- 5: Subjects with an indication, as stated by a Gastroenterologist, for the eradication of *Helicobacter pylori* with a *Helicobacter pylori* eradication therapy as follows: Proton Pump Inhibitor (pantoprazole 40 mg twice daily), clarithromycin 500 mg-twice daily, amoxicillin 1000 mg twice daily, for 14 days.
- 6: For female: If of child bearing potential, female subjects must be using or complying with one of the following medically approved methods of contraception such as, but not exclusively:

a) Oral birth control pills (at least 1 full monthly cycle prior to study product administration);
b) Intra-uterine device (IUD);
c) Double barrier methods (such as condoms and spermicide);

OR Female subject must be postmenopausal for at least 12 months prior to trial entry or surgically sterile (i.e. hysterectomy, bilateral oophorectomy or bilateral tubal ligation).

- 7: Subjects who could and were willing store a maximum of 32 study product bottles (100g each) at any one time in his/her fridge (between +2°C and +8°C).
- 8: Subjects who accepted an alimentary restriction of products including yoghurts, fermented dairy products with probiotics, over-the-counter medication containing probiotics, starting at Day 0 until Last study visit (at least 42 days).
- 9: Subjects who appreciated dairy products and multi-fruit flavour and who were willing to consume 2 bottles per day of study product for 28 consecutive days.
- 10: Subjects who were willing to provide stool and blood samples throughout the duration of the study.
- 11: Subjects who were willing to complete ePRO and had access to internet.

Exclusion criteria

General:

- 1: Female subjects with a positive pregnancy test (based on serum test), or planning to become pregnant during the study or breast-feeding women.

- 2: Subjects enrolled in another interventional clinical study in the last 4 weeks or in an exclusion period following participation in another clinical trial.
- 3: Subjects who reported a foreign travel in the previous 7 days or who planned a foreign travel during the study in a country at risk for contracting diarrhea, including Asia (except for Japan), Middle East, Africa, Mexico, and Central and South America.
- 4: Subjects not able to answer questionnaires by writing whatever the reason.
- 5: Subjects with a loss of personal liberty, by administrative or judicial decision.
- 6: Subjects who were major but with a legal guardian.
- 7: Subjects in a situation which in the investigator's opinion could interfere with optimal participation in the present study or could constitute a special risk for the subject.
- 8: Subjects who had a history of alcohol abuse in the 6 months preceding the study.

Symptoms:

- 9: Subjects having diarrhea within the preceding 4-weeks (defined as any episode of stool of 5–7 types in Bristol Stool Scale).

Medical history:

Global

- 10: Subjects with severe life-threatening illness.
- 11: Subjects presenting a severe evolutive or chronic pathology (e.g. cancer, tuberculosis, Crohn's disease, cirrhosis, multiple sclerosis, Type I diabetes…).

Immune disorders

- 12: Immune-suppressed subjects (for congenital reasons or acquired due to HIV infection, malignancy or its treatment, steroids – endogenous excess or exogenous, post- transplantation or patients receiving cyclosporin, or any clinical condition compromising the patient's immune function).

Gastro-intestinal pathologies:

- 13: Subjects with at least one alarm feature including bleeding, anemia, unexplained weight loss, dysphagia, odynophagia, recurrent vomiting, previous malignancies in the gastrointestinal tract.
- 14: Subjects with benign peptic ulcer or pre-malignant or malignant lesions based on gastroscopy exams within the context of *H. pylori* detection.

Note

- ➢ Duodenal ulcer was not a non-inclusion criteria.
- ➢ For all subjects presenting at least one dyspeptic symptom (i.e, bothersome postprandial fullness or early satiation or epigastric pain), a gastroscopy is proposed systematically.
- 15: Subjects presenting an infection of the of the gastrointestinal tract.
- 16: Subjects presenting a severe evolving or active pathology of the gastrointestinal tract such as cancer, inflammatory bowel syndrome, inflammatory bowel disease, Crohn's disease or ulcerative colitis, diverticular disease, biliary disease, chronic liver disease (based on blood transaminase analysis), any clinical condition affecting the pancreas including acute and chronic pancreatitis, history of metabolic disease (malabsorption, celiac disease) with the exception of dyspepsia and infection related to *Helicobacter pylori*.

- 17: Subjects with any past severe gastro-intestinal or metabolic pathology with the exception of appendicitis.
- 18: Subjects with a history of *Hp* eradication therapy.

Cardiac, renal and surgery issues

- 19: Subjects with cardiac insufficiency.
- 20: Subjects with a history of angina pectoris or other clinically significant cardiac disease.
- 21: Subjects with artificial heart valves (cardiac valvular prosthesis).
- 22: Subjects with a history of endocarditis.
- 23: Subjects with a history of rheumatic fever.
- 24: Subjects with a congenital heart defect.
- 25: Subjects with non-controlled hypertension.
- 26: Subjects having had any surgery or intervention requiring general anaesthesia in the last 4 weeks, or who had planned surgery or an intervention requiring general anaesthesia in the course of the study.
- 27: Subjects who had any dental surgery in the last 4 weeks, or that had planned dental surgery in the course of the study.
- 28: Subjects who had gastro-intestinal surgery in the last 3 months or planned surgery in the course of the study.
- 29: Subjects with renal insufficiencies based on blood creatinine analysis.

Treatments:

- 30: Subjects with allergy or hypersensitivity against the medication for the *Hp* eradication therapy.
- 31: Subjects with antibiotic or intestinal antiseptic treatment during the previous 2 months.
- 32: Subjects with H2-receptor antagonists or PPI treatment in the last 2 weeks.

In case of use of the above treatments in the past two weeks, the subjects were asked to stop the treatment and come back 2 weeks later for *Hp* infection diagnosis and inclusion in the study (unless ineligible for other criteria).

- 33: Chronic use of laxatives or anti-diarrheal.

In case of use of the above treatments in the past two weeks, the subjects were asked to stop the treatment and come back 2 weeks later for inclusion in the study.

- 34: Subjects currently receiving immunosuppressants or chemotherapy.
- 35: Subjects taking any treatment aimed at weight management or any form of treatment likely to interfere with metabolism or dietary habits.

Dietary issues

- 36: Subjects with any diagnosed food allergy.
- 37: Subjects with allergy or hypersensitivity to any component of the study products (e.g.: allergy or hypersensitivity to milk proteins or lactose).
- 38: Subjects with eating disorders (anorexia, boulimia).
- 39: Subjects with special medicated diet (due to obesity, anorexia, metabolic pathology).
- 40: Subjects under artificial nutrition in the last 2 months.

Breath Test

[13]C-Urea Breath Test was carried out using HeliFANplus device (Fisher Analysen Instrumente GmbH) [1], before and 30 minutes after ingestion of an oral substrate dose of 75mg of [^{13}C] urea (99%), dissolved in 250mL of an acidic drink .

Fecal sample collection

A total of 558 fecal samples was collected in the study from 135 (67 in Test and 68 in Control group) subjects at 4 time points (Figure 1). Fresh fecal samples were collected and kept up to 24h at 2–8°C in anaerobic conditions (Genbag Anaerobic, Biomerieux), before being aliquoted and stored at −80°C. All samples were then processed for total bacterial count (flow cytometry) and for DNA extraction in view of microbiota profiling. For SCFA, calprotectin, and Test product strains viability analyses, samples from a subgroup of subjects were analyzed.

Clostridioides difficile detection in feces:

Test for *Clostridioides difficile* (*Cd*) in feces followed a two-step strategy as recommended by the European clinical guidelines [2]. Detection was based on Glutamate dehydrogenase (GDH) and *Cd*-Toxin A and B tests (*Cd*. Quick Check Complete ®, Abbott, US). In case of GDH-/Toxin+ or GDH+/Toxin- results, Polymerase Chain Reaction (PCR) test was used to confirm the presence or absence of a toxigenic strain. *Cd* detection tests were performed in stools collected at day 0, day 28 and in case of AAD (in two samples, one collected on the first day of AAD and one after the end of AAD, i.e. after two consecutive days with stools with BBS type <5).

Quantification of total and viable Test product strains in feces

Strain quantification was performed in 48 subjects, including 40 subjects randomly selected from the FAS population and the 8 subjects who reported an AAD-definition 2. Results are expressed as Total cell count (both live and dead cells), Viable cell count, Viability loss (Total cell count – Viable cell count) and Viability rate ([Viable cell count/Total cell count] x 100) at each planned visit. Total count of three strains from Test product was assessed using a real time quantitative PCR (qPCR) while the count of viable strains was determined through a qPCR combined with propidium monoazide labelling (PMA, Sigma Aldrich, France), that can differentiate live from dead bacteria. Each fecal sample was analyzed in three conditions: (1) qPCR without PMA treatment for total bacteria, (2) PMA-qPCR for viable bacteria and (3) qPCR heat treatment at 95°C/20 min and PMA treatment for PMA efficacy control. PMA was added to 2mg of feces at a final concentration of 100 μM, incubated 5 min in dark at room temperature (RT) with occasional mixing. All samples were light exposed for 15 min, mixed every 3 min at RT using a PhAST Blue Photoactivation System (Geniul, Spain). DNA extraction was performed as previously described [3]. PCR reactions contained an internal control for presence of any PCR inhibition (Eurogentec, Belgium). qPCR run was performed on a 384-well plate (Eppendorf®) with triplicate wells for each sample. qPCR reactions included 2 × QuantiFast Multiplex PCR Mastermix (QIAGEN, France), 1 μL extracted fecal DNA, 500 nM for each primer [3] and 200 nM for each Taqman probe. qPCR amplification was programmed with an initial denaturation at 95 °C for 5 min, followed by 40 cycles of 95 °C/30 s and 60 °C/30 s.

Gut Microbiota:

Flow cytometry

Sample preparation and flow cytometric analysis of the samples was done according to Vandeputte et al. with some minor modifications [4]. For cell counting, 0.1g of fecal sample was weighed inside a sterile falcon tube using an analytical balance. To this

material, 10 mL of sterile PBS was added, and the samples were vortexed thoroughly. Afterwards, the samples were stored for at least 2 hours in the fridge (4°C) to allow proper homogenization of the samples. Subsequently the samples were again vortexed thoroughly. Upon complete homogenization the samples were diluted 100 times in sterile PBS and filtered through a sterile filter (pore size 5 μm). Finally, 50 μL of these 100 times diluted samples was added to 445 μL sterile PBS. The microbes present in these final dilutions were stained with SYTO 24 green fluorescent nucleic acid stain by adding 5 μL of 0.1 mM-stock solution of SYTO 24 to the samples. Finally, the samples were incubated in the dark for 15 min at 37°C prior to flow cytometric analysis. Stained samples were analyzed on a BD FACSVerse. Proper PMT voltage, threshold, and gating settings separated the stained microbes from sample debris and signal noise. The samples were run at medium flow rate (60 μL/min) and events were recorded in a SSC-H:FITC-H dot plot. A threshold of 200 was applied on the FITC-H channel. After flow cytometric analysis, data were analyzed and processed in Flow jo V10.

DNA extraction, 16S and ITS2 sequencing

DNA was extracted from 200 mg frozen stool using the standardised Protocol Q, a protocol that is based on chemical and mechanical lysis (Fastprep® FP120 [ThermoSavant]), and that was identified as the most efficient in the lysis of Gram-positive bacteria and reproducible out of 21 protocols [5]. 50 ng of DNA was amplified following the 16S Metagenomic Sequencing Library Illumina 15044223 B protocol (ILLUMINA) using V3-V4 primers for 16S rRNA gene [6] and ITS2 [7]. The samples were loaded into flow cells in an Illumina MiSeq. 300PE Sequencing Platform in accordance with the manufacturer's instructions. 539 fecal samples were kept for downstream 16S rRNA analyses (135 subjects, (67 in Test and 68 in Control group) for Control) and 374 samples for ITS analyses (97 subjects, $N = 49$ for Test and $N = 48$ for Control).

Bioinformatic analysis

Raw sequences, forward and reverse, were merged in order to obtain the complete sequence using the 'pear v0.9.6' software. The amplification primers from the sequences obtained in the sequencing step were trimmed to reduce the bias in the annotation step, with 'cutadapt v 1.8.1' and parameters by default. Once the primers removed, sequences smaller than 200 nucleotids were removed from the analysis. After obtaining the clean complete sequences, a quality filter was applied to them to delete poor quality sequences. Those bases in extreme positions that did not reach Q20 (99% well incorporated base in the sequencing step) or a greater quality score were removed. Subsequently, sequences whose average quality did not surpass the Q20 threshold, as a mean quality of the whole sequence, were also deleted. The resulting sequences were inspected for PCR chimera constructs that may occur during the different experimental processes. Chimeras were removed from further analysis. Analyses were performed using QIIME (v. 19). After filtering for quality, a mean of 99,437 ± 36,973 sequences per sample were retained. Reads were clustered into operational taxonomic units (OTUs; 97% identity threshold) using VSEARCH, and representative sequences for each OTU were aligned and taxonomically assigned using the SILVA database (v. 119) and the Unite database for ITS (v. 7.2). To characterize diversity, rarefaction was used to obtain 22,000 sequences per sample. Alpha diversity (within samples) was assessed using Shannon index and Simpson's Reciprocal index for 16S rRNA and using Shannon index for ITS. The ITS/16S ratio was computed for Shannon index [8]. Beta diversity was represented using Bray-Curtis dissimilarity and UniFrac distances (weighted and unweighted) for 16S rRNA and Bray-Curtis dissimilarity for ITS.

SCFA and Calprotectin dosag*e*

SCFA and Calprotectin were analyzed in subgroups of the last 61 and 73 subjects randomised respectively. Calprotectin concentration in feces was measured by ELISA (PhiCal Calprotectin ELISA, Immungiagnostik AG, Germany). SCFA concentration in stools (per gram of dry feces) and in serum was measured by headspace gas chromatography mass spectrometry (GC-MS) (column Rx-624Sil MS, RESTEK, France).

Safety parameters

Safety assessment was performed by following vital signs (systolic and diastolic blood pressure, heart rate, body temperature) and anthropometry (body height, weight and Body Mass Index) at days −45 (up to), 0, 7, 14, 21, 28 and 42, blood analyses of partial hemogram (red blood cells, leucocytes, haemoglobin, haematocrit, neutrophils, eosinophils, basophils, lymphocytes, monocytes and platelets), glucose, insulin, Insulin Resistance (HOMA-IR), cholesterol (total, HDL, LDL) and triglycerides at days 0, 28 and 42, hepatic enzymes (ALT, AST, γGT) and creatinine at days −45 (up to), 0, 28 and 42. The occurrence and the number of spontaneously reported adverse events were assessed throughout the study as well as their relationship to the study product or the *Hp*-treatment, their intensity, seriousness, and the appropriate action taken (interruption of product intake or *Hp*-treatment or subject withdrawal).

Procedure

Subjects underwent a physical examination and vital signs were recorded at each visit. At V2, subjects reported physical activity habits on a usual week by answering the Short last 7 days self-administered version of the Physical Activity Questionnaire (IPAQ) (revised august 2002 version) [9] and smoking habits on the last month (expressed in number of smoked cigarettes per week, using the following equivalences: 1 pipe = 5 cigarettes; 1 small cigar or cigarillos = 2 cigarettes; 1 big cigar = 5 cigarettes). Subjects dietary habits during the past month was reported at V2 and V6 at investigation site through a Food Frequency Questionnaire (FFQ) [10], adapted for diet reporting of the past month. FFQ outputs were converted in daily amounts of nutrient intake using software developed by the German Institute of Human Nutrition (DIFE), Potsdam. Subject alcohol consumption was reported weekly at all visits and converted to the average number of alcohol units per week (one unit= 10 grams of pure alcohol). In a personal e-diary, through electronic Patient Reported Outcomes (ePRO) software (Patient Cloud, Medidata), subjects daily reported their compliance with the *Hp* eradication therapy, with the study product consumption and with the dietary restrictions, in the corresponding periods, as well as any adverse event, concomitant medication and nutritional supplement intake from V2 to V7. Between V2 and V6, subjects also used ePRO for daily reporting of all passages of stool and their consistency (from 1 to 7 as based on BSS [11] and of main GI symptoms, and for filling GSRS questionnaire [12] once a week. E-diary was remotely monitored for completion by the investigator or authorized staff on a daily basis and weekly during visits. ^{13}C-urea breath test was performed at V1 for *Hp* infection diagnosis process and at V7 (not earlier than 28 days after the last *Hp* treatment dose) to check *Hp* eradication. The study was performed in accordance with the protocol with no change to trial outcomes during the course of the trial. Source data verification was performed by monitors appointed by a third-party contractor (Pharmaceutical Product Development, llc).

Data Monitoring Committee and Interim analysis

DMC organisation

DMC was composed of five independent experts in gastro-enterology and treatment of *Hp* infection, in pediatrics, gastro-enterology, and probiotics, in public health, infectious diseases and management of nosocomial infections, in development of PRO instruments

and in Statistics. Details of DMC organization, responsibilities, data flow, decision algorithm and communication were provided in a DMC charter that was signed together with final Statistical Analysis Plan before the interim database lock. The study staff (except the product supply supervisor) including the sponsor statistician remained blinded until the end of the study.

Stopping rules at interim analysis

Different scenarios and decision rules were presented to the DMC members to guide the recommendation to stop or to continue the study after the interim analysis. For each scenario, recommendations were based on a sample size re-estimation through the adaptative approach of the Conditional Power (CP) proposed by Mehta and Pocock. For each AAD definition, CP were computed based on data observed on the first 104 evaluable subjects for primary efficacy outcome (Definitions 1 and 2 of AAD occurrence). The following decisions algorithm was carried out for each of the Definition (X = 1 or 2) defined:

- If CP < 30% then the primary outcome for Definition X of AAD was futile
- If 30% ≤ CP ≤ 60% then the sample size required to increase the CP to 80% was computed and the study could continue as a POC for Definition X with a sample size minimum of 550 evaluable subjects
- If CP > 60% then the study continued as a Proof of Efficacy (POE) for Definition X of AAD with a sample size of 550 evaluable subjects to reach 80% power.

Interim analysis

The interim analysis was performed when 104 subjects could be evaluated, and was reviewed by the DMC. As only one subject experienced AAD episode on AAD definition 1, the decision algorithm was based on AAD definition 2. Finally, Definition 2 of AAD occurrence was too low and primary outcome judged as futile. No sample size re-estimation was made. The study sponsor followed the DMC recommendation to discontinue the trial after interim analysis due to futility of clinical efficacy endpoints. As recruitment continued during the interim analysis, 136 subjects were randomised in total for which all analyses had been completed and are presented here.

Statistical analysis

SCFA, Calprotectin, quantification of Test product strains

The *Hp* treatment effect on change of biological parameters was assessed with a repeated measures linear mixed model. For Calprotectin and each SCFA, three covariance structures (unstructured, autoregressive and compound symmetry) were investigated and the best model with the lowest Akaike Information Criteria was retained. Normality of residuals was verified with skewness and kurtosis, for both statistics a [−2 ; 2] interval was considered as acceptable to state as the normality. If the assumption of normality was not met, a log-transformation was applied, and the same repeated linear model was used. Due to exploratory context, no alpha adjustment for multiple testing strategies was planned. For the quantification of Test product strains in feces, for each strain quantified, the effect of time points (V4, V6 and V7) on total and viable cell count was assessed using a non-parametric Friedman test. A 2 by 2 comparison (Wilcoxon signed-rank test) for time points V4 vs. V6 and V6 vs. V7 was performed with a Bonferroni correction for multiple testing. Due to a higher rate of missing data at V7 observed for viability loss and viability percentage, the effect of time points (V4 and V6) was assessed with a Wilcoxon test.

Gut microbiota and microbiota

For beta diversity, associated tests of multivariate homogeneity of group dispersion were computed as heterogeneity can bias significance in PERMANOVA. Principal

Coordinate Analyses were performed to complement PERMANOVA. An intra-subject analysis was performed comparing V4, V6, and V7 with the corresponding baseline (V2) using Mann-Whitney U test. DESeq2 model was set to identify differentially abundant genera between Group and Visit while considering the microbiota composition at baseline (V2) and controlling for differences within individuals, as detailed in the DESeq2 tutorial. For QMP, the normalization step was thus omitted, and the model built on the QMP-normalized values divided by 100 for R to deal with large counts. *P*-values were adjusted for each parameter in each dataset and alpha risk was set to 0.05.

Supplementary Results

Clinical outcomes

Results on alternative AAD definition, time to first event and AAD duration (Definition 1 and 2) did not show any significant difference between groups either (data not shown). A slightly higher mean number of days with GI symptoms was observed in the Test as compared to the Control group (mean (SD) of 9.5 (8.0) vs. 8.4 (8.5) days), mainly due to bloating, but the difference was low in proportion and no conclusion can be driven on any product effect (**Table S2**). No relevant difference between groups was observed either for the Total GSRS score or the GSRS scores by dimension at each kinetic point (**Figure S2**) or as a change from baseline at each visit. Total and dimensions scores globally decreased, albeit slightly (<1-point score evolution), all along the study between V2 and V6, with the same trends in both groups. Diarrhea dimension score was the only one showing a slight increase between baseline (V2) and V3 or V4, similarly in both product groups.

Test product strains viability in feces

The viability of the three strains *Lp* CNCM I-3689, *Lr* CNCM I-3690 and *Lp* CNCM I-1518 was assessed in the feces of 48 subjects (25 in Test, 23 in Control group) (**Figure S3**). Some samples from Control group at V4 or V6 and from Test group at V2, showed a detectable signal in Total cell count of strains, mainly *Lp* CNCM I-1518, in less than 5% of the tested samples overall, in the absence of Test product intake, representing therefore a low proportion of possible false positive detection. At V4, after 2 weeks of Test product intake and concomitant *Hp*-treatment, total cell (live and dead) counts reached 8.1 to 8.3 $\log_{10}$ cells/g feces depending on the strain. Viable cell count ranged from 6.5 to 7.1 $\log_{10}$ /g feces, with the highest count found for the *Lp* I-1518 strain (**Figure S3**). The associated viability rate varied accordingly from 2.2% for *Lr* I-3690 to 2.3% for *Lp* I-3689 and 9.4% for *Lp* I-1518, corresponding to a viability loss of 1.7, 1.6 and 1 $\log_{10}$ respectively. At V6, for the three strains, at the end of Test product consumption period, no significant change was observed for either the total or viable cell count as compared to V4 when *Hp* treatment ceased ($p > 0.05$). The viability rate and resulting viability loss were also not significantly different between V4 and V6 for *Lr* I-3690 and *Lp* I-3689 whereas a statistically significant decrease of the viability rate (from 9.4 to 3.5%, Friedman Rank Test $p < 0.05$) and increase of viability loss (from 1 to 1.5 $\log_{10}$, Friedman Rank Test $p < 0.01$) was observed for *Lp* I-1518. At V7, two weeks after the end of the last Test product intake, both total and viable bacteria count significantly decreased for all three strains as compared to V6 (Wilcoxon signed-rank, $p < 0.001$) to an undetectable level (LOQ), except for *Lr* I-3690 in 5 and 2 subjects (20% and 8% of subjects) and for *Lp* I-3689 strain in 1 and 1 subject (4% and 4% of subjects) respectively.

Gut mycobiota response to Hp treatment

At baseline, *Ascomycota* was the major phylum (median of 60% of total mycobiota). Most dominant assigned fungal genera included *Saccharomyces, Penicillum, Candida, Aspergillus and Torulaspora*. Beta-diversity measured by Bray-Curtis dissimilarity showed that there was a global shift in mycobiota composition at V4 compared to baseline (PERMANOVA adjusted $p = 0.005$) and a gradual but incomplete recovery (PERMANOVA, adjusted $p = 0.015$) **(Figure S6).** Test and Control were not different at any timepoint. Intra-subject diversity as distance to baseline was not different between groups at any timepoint or along time.

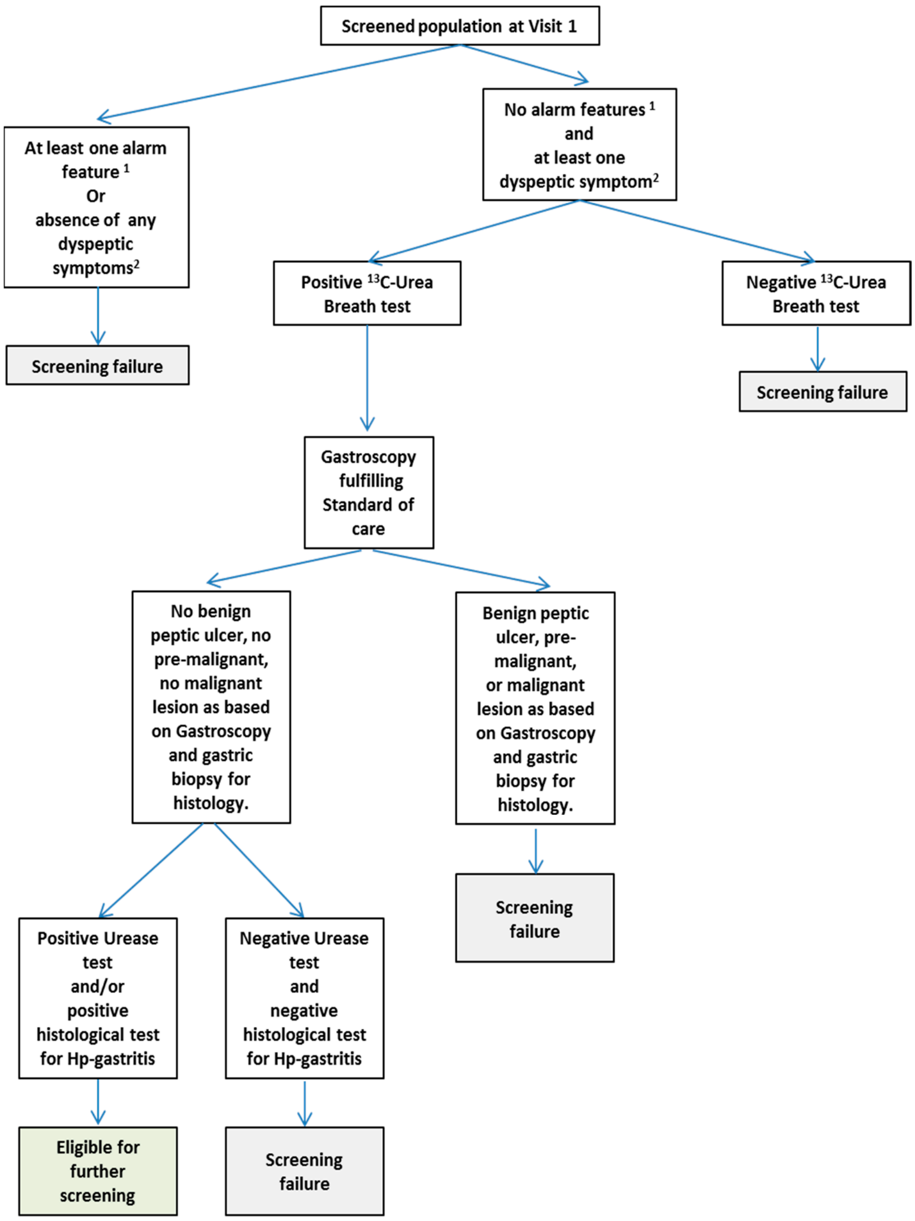

Figure S1. Decision tree for diagnosis of *Hp* infection and subject screening. [1] : Alarm features include: bleeding, anemia, unexplained weight loss, dysphagia, odynophagia, recurrent vomiting, malignancies in the gastrointestinal tract. [2] : Dyspeptic symptoms : bothersome postprandial fullness, early satiation, epigastric pain.

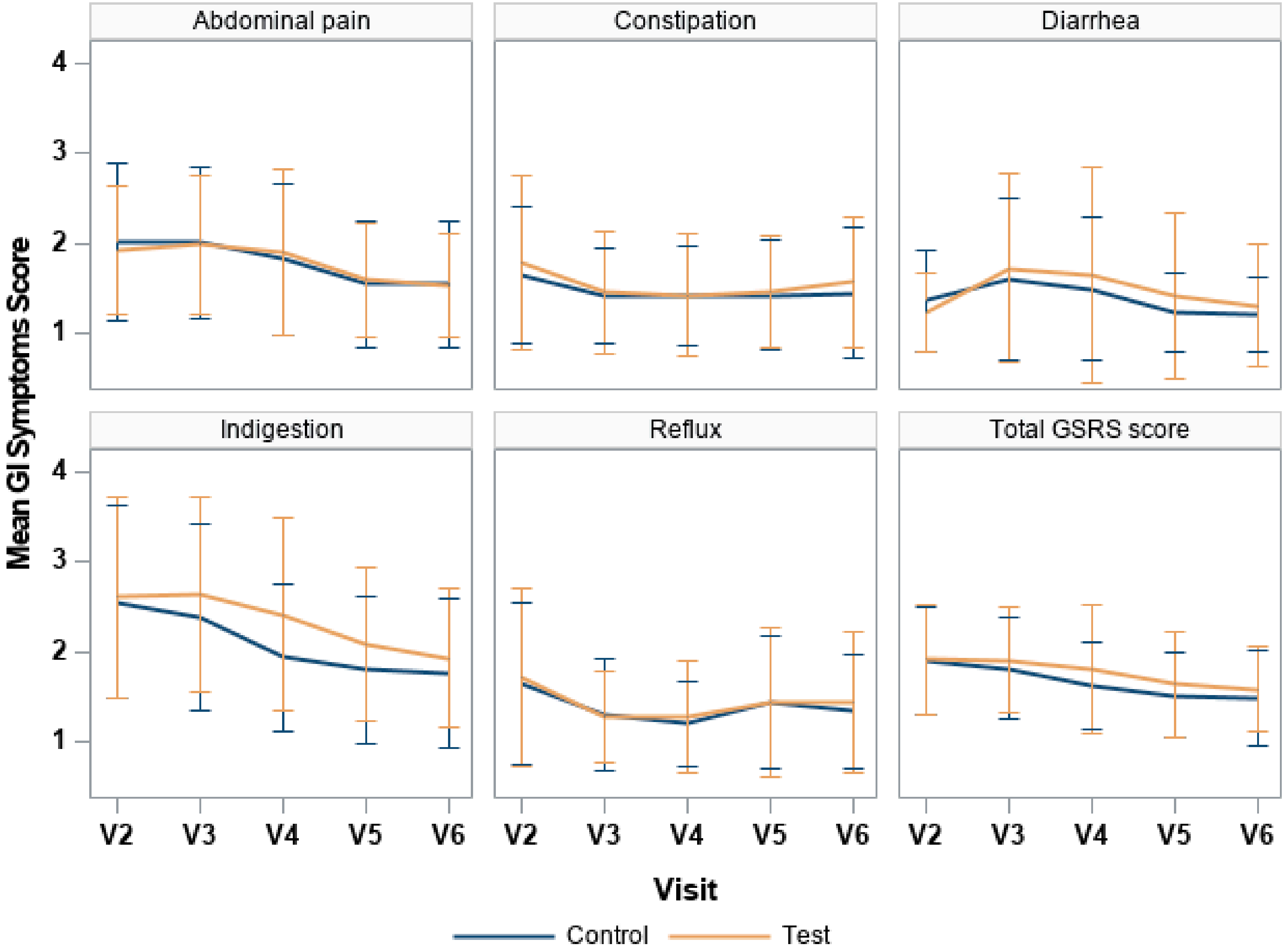

Figure S2. GSRS. Total GSRS score and scores by each GSRS dimension per visit.

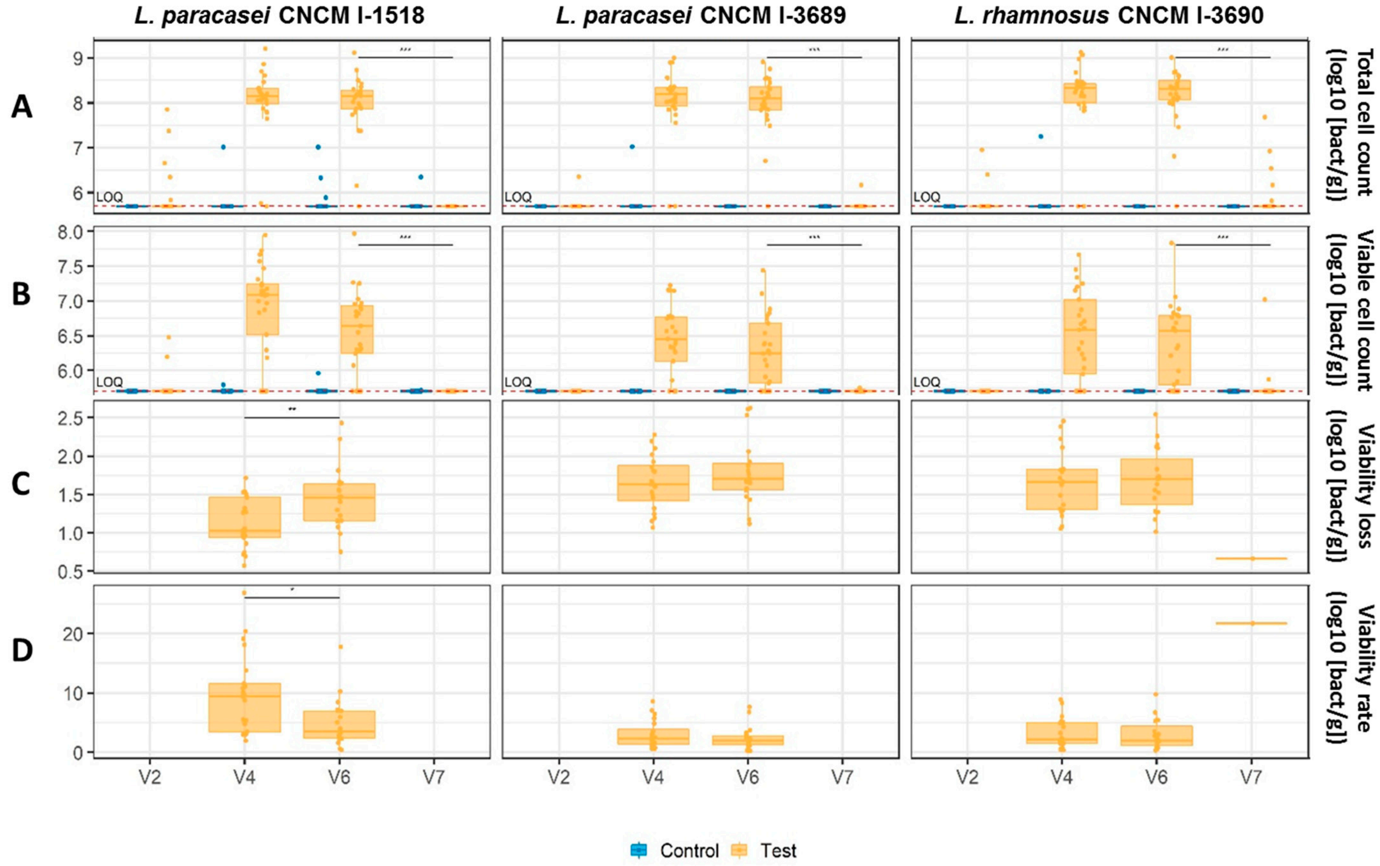

Figure S3. Quantification of Test product strains in feces. (A) Total (live and dead) cell count, (B) Viable cell count, (C) Viability loss (Total cell count – Viable cell count) and (D) Viability rate ([Viable cell count/Total cell count] x 100), per visit. For Total and Viable cell count, data below the limit of quantification (LOQ) were imputed at the LOQ (5.7 log_{10} [bacteria cell number/g feces]). Imputed values are used for V4, V6, V7 data and non-imputed values for analyses of changes between visits (V6–V4, V7–V6). For Viability loss and Viability rate, when Total or Viable cell counts were below LOQ, data were not imputed and considered as missing. P-values are given for comparison between visits in Test group and after Bonferroni correction for Total and Viable cell count variables (*$p \leq 0.05$, ** $p \leq 0.01$; ****$p \leq 0.0001$). Total cell count, Viable cell count and Viability loss are expressed in log_{10} [bacteria cell number/g feces])(log_{10} [bact/g]).

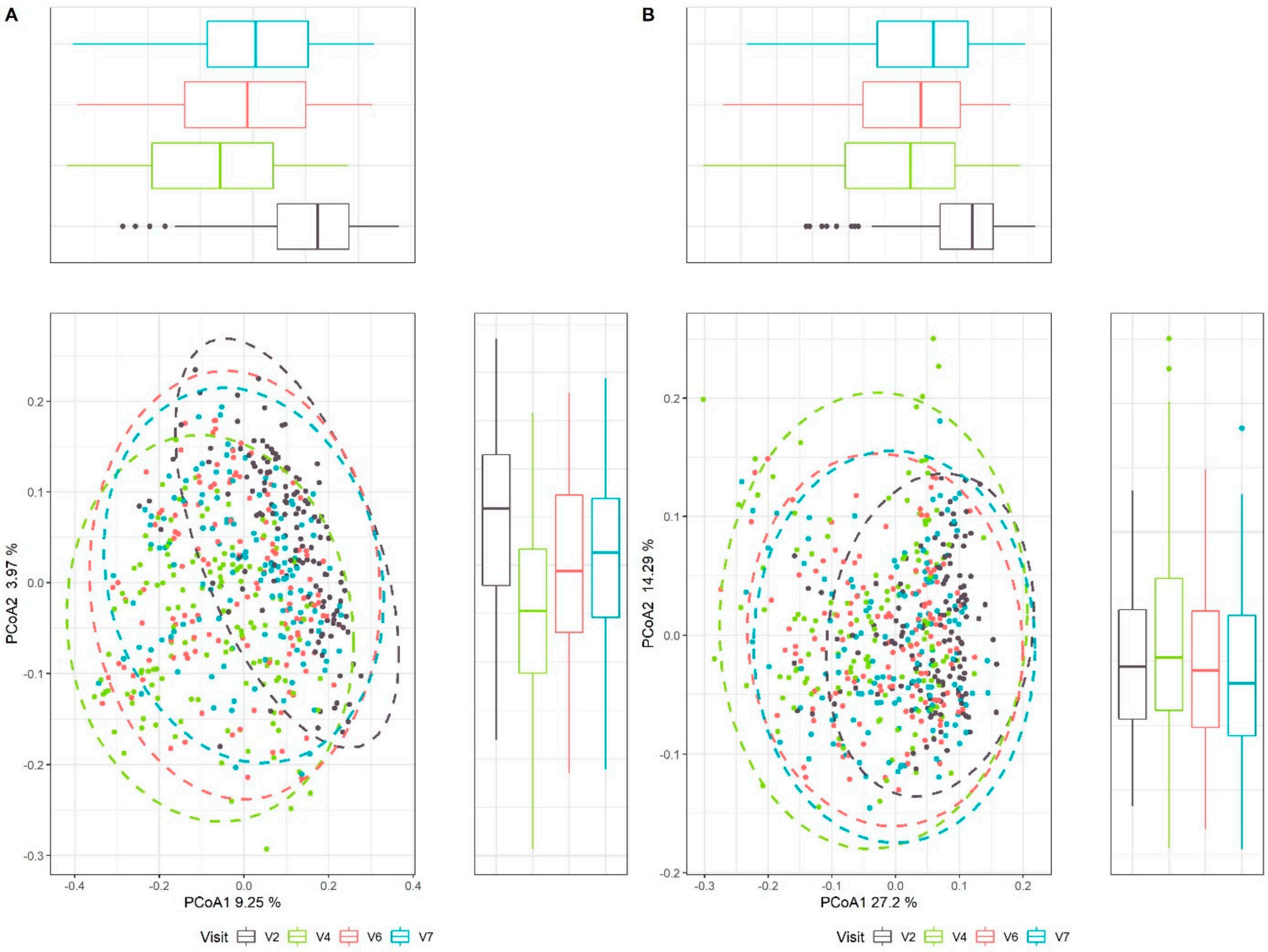

Figure S4. UniFrac beta-diversity. (A) Principal Coordinate analysis (PcoA) of unweighed UniFrac. (B) weighted UniFrac.

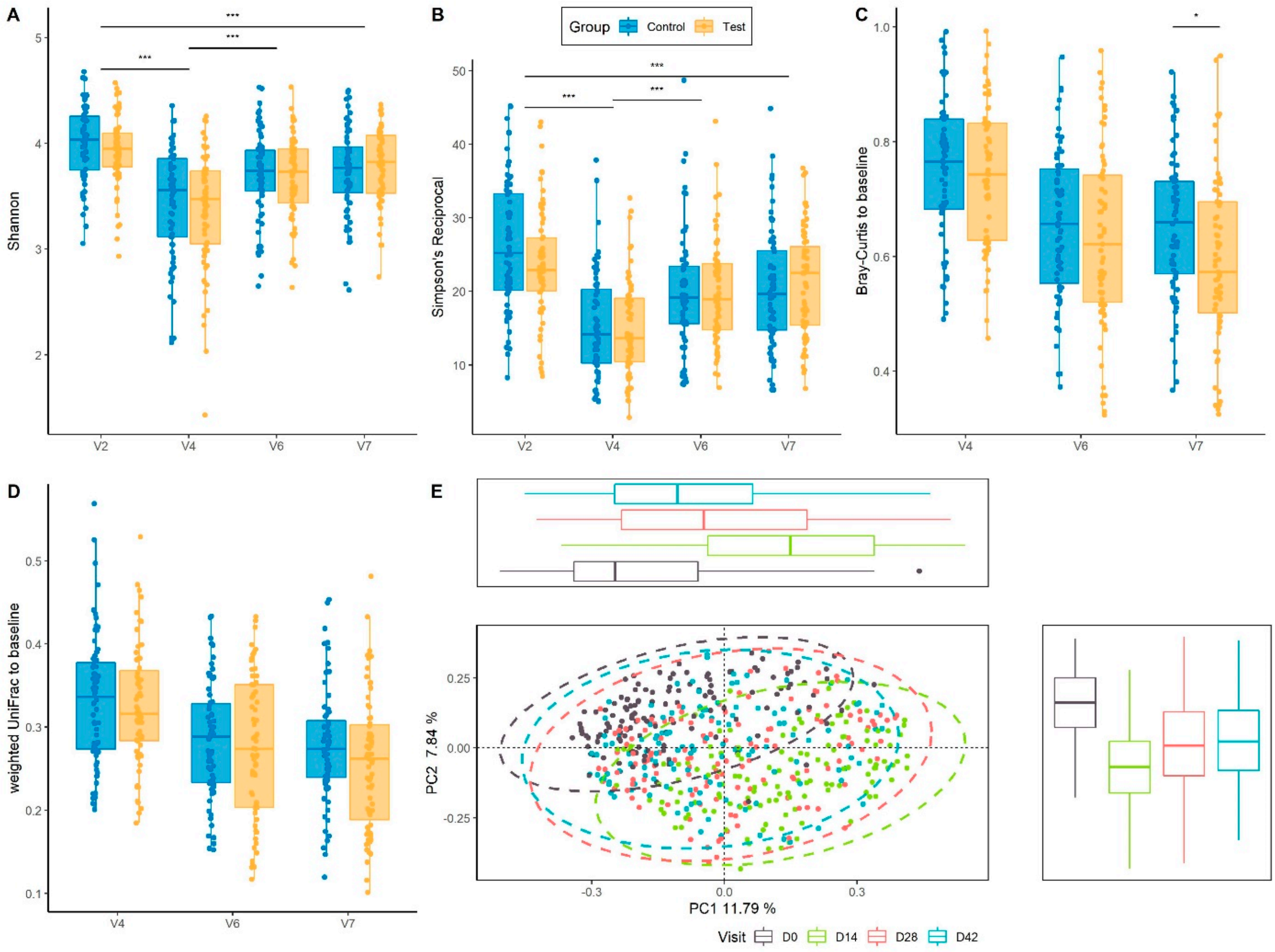

Figure S5. Global gut microbiota response to *Hp* treatment by Quantitative Microbiome Profiling. (A) Alpha-diversity assessed by Shannon index and (B) Alpha-diversity assessed by Simpson reciprocal (C) Intra-subject distance to baseline in Test and Control groups across the study based on Bray-Curtis dissimilarity (D) Intra-subject distance to baseline in Test and Control groups across the study based on Weighted UniFrac distance (E) Principal Coordinate analysis (PcoA) of Bray-Curtis dissimilarity. * $p < 0.05$; ** $p < 0.01$; *** $p < 0.001$.

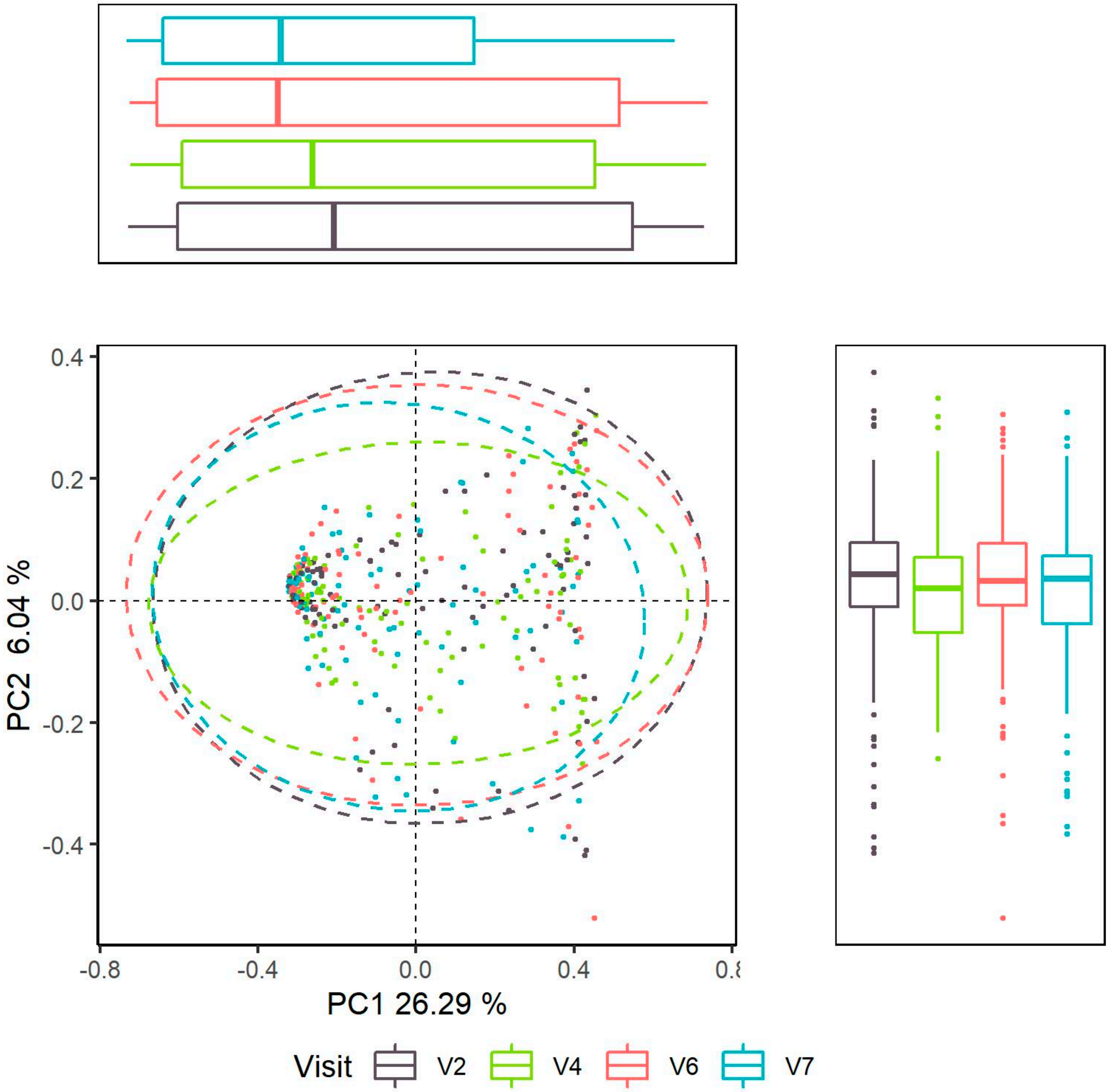

Figure S6. Global gut mycobiota response to *Hp* treatment. Principal Coordinate analysis (PcoA) of Bray-Curtis dissimilarity.

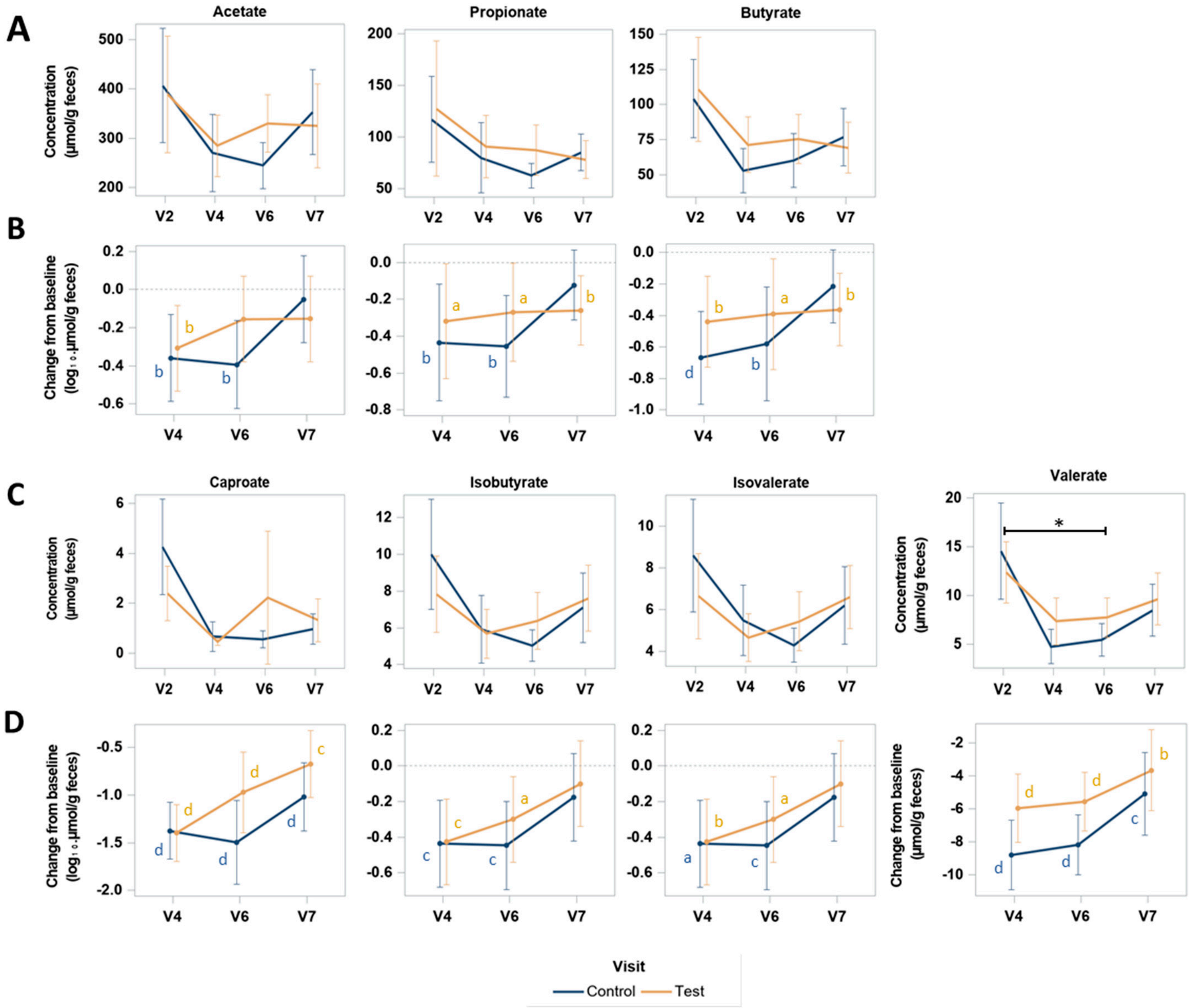

Figure S7. Quantification of individual fecal SCFA. Concentration (A,C) and change from baseline (B,D) of SCFA: acetate, propionate, butyrate (A,B), caproate, isobutyrate, isovalerate, valerate (C,D). P-values are provided according to Student test: * $p < 0.05$ for comparison between groups of the change of valerate concentration from V2 to V6; [a] $p < 0.05$, [b] $p < 0.01$; [c] $p < 0.001$; [d] $p < 0.0001$, for comparison within group of the change from V2 at each visit. SCFA concentrations are expressed in µmol/g of dry feces.

Table S1. Bacterial strain counts in Test product during the shelf life.

Strains	Shelf Life	Mean (CFU/g)[1]	Min (CFU/g)	Max (CFU/g)
Lacticaseibacillus paracasei CNCM I-1518	Day 3	2.85 x 10^8	1.50 x 10^8	5.00 x 10^8
	Day 42	1.85 x 10^8	1.20 x 10^8	3.90 x 10^8
Lacticaseibacillus paracasei CNCM I-3689	Day 3	2.26 x 10^7	5.00 x 10^6	4.30 x 10^7
	Day 42	1.82 x 10^7	6.00 x 10^6	3.90 x 10^7
Lacticaseibacillus rhamnosus CNCM I-3690	Day 3	1.34 x 10^8	7.20 x 10^7	2.20 x 10^8
	Day 42	3.40 x 10^7	1.20 x 10^7	1,00 x 10^8
Streptococcus thermophilus (CNCM I-2773, CNCM I-2835, CNCM I-2778)	Day 3	5.56 x 10^8	3.70 x 10^8	7.50 x 10^8
	Day 42	5.44 x 10^8	2.60 x 10^8	8.10 x 10^8
Lactobacillus delbrueckii subsp bulgaricus CNCM I-2787	Day 3	3.58 x 10^6	1.00 x 10^5	2.00 x 10^7
	Day 42	5.60 x 10^2	1.00 x 10^1	5.40 x 10^3

[1] : Means were calculated as derived from measures carried out in each of the 18 batches of product used in the study. Counts were measured for each of the bacterial strains present in the Test product, at the start (day 3) and end (day 42) of the authorized storage period (shelf life).

Table S2. Number of days of GI symptoms.

		Test (*N* = 68)	Control (*N* = 68)
Number of days with GI symptoms[1]	Mean (SD)	9.5 (8.0)	8.4 (8.5)
	Min ; Max	0 ; 28	0; 28
Number of days with diarrhea[2]	Mean (SD)	0.2 (1.2)	0.1 (0.5)
	Min ; Max	0 ;8	0 ; 3
Number of days with abdominal pain	Mean (SD)	2.4 (3.4)	2.4 (3.6)
	Min ; Max	0 ; 18	0 ; 13
Number of days bloating	Mean (SD)	8.1 (8.0)	6.7 (8.1)
	Min ; Max	0 ; 28	0 ; 28
Number of days nausea	Mean (SD)	0.8 (1.4)	1.4 (2.8)
	Min ; Max	0 ; 6	0 ; 14
Number of days vomiting	Mean (SD)	0.0 (0.1)	0.1 (0.4)
	Min ; Max	0 ; 1	0 ; 2

[1] : Total cumulative number of days with GI symptoms between V2 and V6. [2] : Based on AAD reporting according to AAD definition 2.

Table S4. Adverse events.

	Test (*N* = 68)		Control (*N* = 68)		All (*N* = 136)	
	Event [1]	*n* (%) [2]	Event [1]	*n* (%) [2]	Event [1]	*n* (%) [2]
AEs	91	42 (61.8)	93	42 (61.8)	184	84 (61.8)
Serious AEs	0	0	1	1 (1.5)	1	1 (0.7)
Severe AEs	0	0	2	1 (1.5)	2	1 (0.7)
AEs unlikely related to study product	38	22 (32.4)	55	30 (44.1)	93	52 (38.2)
AEs causing study product interruption	3	1 (1.5)	0	0	3	1 (0.7)
AEs related to *Hp* eradication treatment	53	28 (41.2)	47	28 (41.2)	100	56 (41.2)
AEs causing *Hp* treatment interruption	7	4 (5.9)	3	3 (4.4)	10	7 (5.1)

[1]: Number of AE. [2]: Number and percentage of subjects with AE. Subject or event in more than one category are counted in each of those categories. Related AE have been categorized as 'Unlikely','Possibly','Probably' or 'Definitely' related. Any events with a missing causality was assumed to be related.

References

1. Braden, B.; Caspary, W.F.; Lembcke, B. Nondispersive infrared spectrometry for 13CO2/12CO2-measurements: a clinically feasible analyzer for stable isotope breath tests in gastroenterology. *Zeitschrift fur Gastroenterologie* **1999**, *37*, 477-481.
2. Crobach, M.J.; Dekkers, O.M.; Wilcox, M.H.; Kuijper, E.J. European Society of Clinical Microbiology and Infectious Diseases (ESCMID): data review and recommendations for diagnosing *Clostridium difficile*-infection (CDI). *Clinical microbiology and infection : the official publication of the European Society of Clinical Microbiology and Infectious Diseases* **2009**, *15*, 1053-1066, doi:10.1111/j.1469-0691.2009.03098.x.
3. Gobert, G.; Cotillard, A.; Fourmestraux, C.; Pruvost, L.; Miguet, J.; Boyer, M. Droplet digital PCR improves absolute quantification of viable lactic acid bacteria in faecal samples. *Journal of microbiological methods* **2018**, *148*, 64-73, doi:10.1016/j.mimet.2018.03.004.
4. Vandeputte, D.; Kathagen, G.; D'hoe, K.; Vieira-Silva, S.; Valles-Colomer, M.; Sabino, J.; Wang, J.; Tito, R.Y.; De Commer, L.; Darzi, Y., et al. Quantitative microbiome profiling links gut community variation to microbial load. *Nature* **2017**, *551*, 507-511, doi:10.1038/nature24460.
5. Costea, P.I.; Zeller, G.; Sunagawa, S.; Pelletier, E.; Alberti, A.; Levenez, F.; Tramontano, M.; Driessen, M.; Hercog, R.; Jung, F.-E., et al. Towards standards for human fecal sample processing in metagenomic studies. *Nature Biotechnology* **2017**, *35*, 1069-1076, doi:10.1038/nbt.3960.
6. Klindworth, A.; Pruesse, E.; Schweer, T.; Peplies, J.; Quast, C.; Horn, M.; Glöckner, F.O. Evaluation of general 16S ribosomal RNA gene PCR primers for classical and next-generation sequencing-based diversity studies. *Nucleic Acids Res* **2012**, *41*, e1-e1, doi:10.1093/nar/gks808.
7. Nash, A.K.; Auchtung, T.A.; Wong, M.C.; Smith, D.P.; Gesell, J.R.; Ross, M.C.; Stewart, C.J.; Metcalf, G.A.; Muzny, D.M.; Gibbs, R.A., et al. The gut mycobiome of the Human Microbiome Project healthy cohort. *Microbiome* **2017**, *5*, 153, doi:10.1186/s40168-017-0373-4.
8. Lemoinne, S.; Kemgang, A.; Ben Belkacem, K.; Straube, M.; Jegou, S.; Corpechot, C.; Chazouillères, O.; Housset, C.; Sokol, H. Fungi participate in the dysbiosis of gut microbiota in patients with primary sclerosing cholangitis. *Gut* **2020**, *69*, 92-102, doi:10.1136/gutjnl-2018-317791.
9. Craig, C.L.; Marshall, A.L.; Sjöström, M.; Bauman, A.E.; Booth, M.L.; Ainsworth, B.E.; Pratt, M.; Ekelund, U.; Yngve, A.; Sallis, J.F., et al. International physical activity questionnaire: 12-country reliability and validity. *Medicine and science in sports and exercise* **2003**, *35*, 1381-1395, doi:10.1249/01.Mss.0000078924.61453.Fb.
10. Nöthlings, U.; Hoffmann, K.; Bergmann, M.M.; Boeing, H. Fitting portion sizes in a self-administered food frequency questionnaire. *J Nutr* **2007**, *137*, 2781-2786, doi:10.1093/jn/137.12.2781.
11. Blake, M.R.; Raker, J.M.; Whelan, K. Validity and reliability of the Bristol Stool Form Scale in healthy adults and patients with diarrhoea-predominant irritable bowel syndrome. *Alimentary Pharmacology & Therapeutics* **2016**, *44*, 693-703, doi:10.1111/apt.13746.
12. Kulich, K.R.; Madisch, A.; Pacini, F.; Piqué, J.M.; Regula, J.; Van Rensburg, C.J.; Újszászy, L.; Carlsson, J.; Halling, K.; Wiklund, I.K. Reliability and validity of the Gastrointestinal Symptom Rating Scale (GSRS) and Quality of Life in Reflux and Dyspepsia (QOLRAD) questionnaire in dyspepsia: A six-country study. *Health and Quality of Life Outcomes* **2008**, *6*, 12, doi:10.1186/1477-7525-6-12.
